# Protocol development to further differentiate and transition stem cell-derived pancreatic progenitors from a monolayer into endocrine cells in suspension culture

**DOI:** 10.1038/s41598-023-35716-1

**Published:** 2023-06-01

**Authors:** Mitchell J. S. Braam, Jia Zhao, Shenghui Liang, Shogo Ida, Nick K. Kloostra, Diepiriye G. Iworima, Mei Tang, Robert K. Baker, Nina Quiskamp, James M. Piret, Timothy J. Kieffer

**Affiliations:** 1 Department of Cellular and Physiological Sciences, Life Sciences Institute, University of British Columbia, Vancouver, Canada; 2 STEMCELL Technologies, Vancouver, BC Canada; 3 Michael Smith Laboratories, University of British Columbia, Vancouver, BC Canada; 4 Department of Chemical and Biological Engineering, University of British Columbia, Vancouver, BC Canada; 5 School of Biomedical Engineering, University of British Columbia, Vancouver, BC Canada; 6 Department of Surgery, University of British Columbia, Vancouver, BC Canada

**Keywords:** Pluripotent stem cells, Stem-cell differentiation, Diabetes

## Abstract

The generation of functional β-cells from human pluripotent stem cells (hPSCs) for cell replacement therapy and disease modeling of diabetes is being investigated by many groups. We have developed a protocol to harvest and aggregate hPSC-derived pancreatic progenitors generated using a commercially available kit into near uniform spheroids and to further differentiate the cells toward an endocrine cell fate in suspension culture. Using a static suspension culture platform, we could generate a high percentage of insulin-expressing, glucose-responsive cells. We identified FGF7 as a soluble factor promoting aggregate survival with no inhibitory effect on endocrine gene expression. Notch inhibition of pancreatic progenitor cells during aggregation improved endocrine cell induction in vitro and improved graft function following implantation and further differentiation in mice. Thus we provide an approach to promote endocrine formation from kit-derived pancreatic progenitors, either through extended culture or post implant.

## Introduction

Type I diabetes manifests when β-cells in the pancreas are depleted, and cannot produce/secrete sufficient insulin, resulting in chronic hyperglycemia. As an alternative to frequent blood glucose measurements and insulin injections following every meal, cell-based therapies have generated significant interest in the field. Cadaveric islet transplantation has been successful in bringing at least temporary insulin independence to most of the treated patients with diabetes and reducing the frequency of hypoglycemia caused by inappropriate insulin dosing, indicating that cell replacement therapies are a viable solution for the disease^1^. However, an insufficient supply of high-quality donor islets and the need for chronic immunosuppression prevent the widespread application of this method. Human pluripotent stem cells (hPSCs) have the potential to generate an unlimited supply of glucose-responsive human pancreatic β-cells and have been an active area for the study of diabetes and its potential treatment^2^.

During in vivo development in both humans and mice, gastrulation of the developing blastocyst forms definitive endoderm tissue and subsequent invagination creates the primitive gut tube^3^. The pancreatic organ bud arises from *PDX1* expressing foregut/midgut tissue, anterior to the *CDX2* expressing intestinal progenitors^4^. From PDX1 + cells, specification into the pancreatic lineage involves the induction of NKX6.1 transcription before pancreatic endoderm designates into more specialized tissue^5^. Patterning of these pancreatic progenitors facilitates segregation into “tip” cells that later develop into the exocrine acinar tissue and bipotent “trunk” cells, which can form the ductal epithelium or, with the suppression of Notch signaling and a subsequent transient wave of neurogenin-3 (NGN3), the endocrine islets^6,7^. By overcoming the repressive threshold set by Notch/Hes1 signaling, cells that commit to an endocrine fate exit the cell cycle and delaminate from the epithelium^8,9^. The co-localization of SOX9 protein with PDX1 during pancreatic budding, indicates that it too is expressed in pancreatic progenitor cells^10^. However, SOX9 and HES1 later colocalize together apart from NGN3 during pancreatic patterning, suggesting that SOX9 is enriched in Notch signal-transducing cells^8,10^. During adult mouse and human pancreas development, SOX9 expression becomes restricted to a sub-population of ductal and acinar cells^10^. Downstream of NGN3 activation, the pan-endocrine marker NEUROD1 mobilizes to maintain endocrine signaling^11,12^. Further signaling designates the endocrine progenitors into specific cell types, with PAX4 driving β-cell and δ-cell emergence and ARX influencing the α-cell and PP-cell pathway^13,14^.

Many protocols have been developed for the derivation of pancreatic progenitors and later endocrine cell types^15–24^. In most β-cell protocols, cells are differentiated stepwise through specific stages resembling definitive endoderm (Stage 1), gut-tube endoderm (Stage 2), posterior foregut (Stage 3), pancreatic endoderm/progenitors (Stage 4), endocrine progenitors (Stage 5), and maturing beta/islet-cells (Stage 6 +). Cells at the pancreatic progenitor stage or later can also be implanted and mature in vivo into functional β-cells that can maintain glucose homeostasis in rodent models of insulin-dependent diabetes^25–27^.

The STEMdiff™ Pancreatic Progenitor Kit contains serum-free media that support the efficient and reproducible generation of pancreatic progenitor cells from mTeSR™1-maintained hPSCs in a 2D monolayer. Upon completion of the 14-day differentiation protocol, cells express key markers of pancreatic progenitor cells, including PDX1, NKX6.1, and SOX9. While certain differentiation protocols involve complete suspension culture through to Stage 6 +^19–22^ or complete monolayer culture through to endocrine/islet-like cells^15,23,27^, other protocols rely on a transition from monolayer culture to suspension culture at the end of Stage 4^18,24,28^. The STEMdiff™ Pancreatic Progenitor Kit generates pancreatic progenitor cells in a monolayer format. Here we formulated a strategy to harvest Kit-derived pancreatic progenitors from 2D culture and aggregate them into relatively uniform spheroids, followed by transition into suspension culture, and continued differentiation toward the pancreatic endocrine lineage. Using a static suspension culture system following aggregation, we were able to generate islet-like clusters with ~ 50% NKX6.1 + /INS + functional β-cells capable of secreting insulin in response to both high glucose and depolarization challenges. By comparing several reported medium formulations, we also determined that FGF7 promotes aggregate survival in shaker suspension culture. In our efforts to drive down *SOX9* expression to improve endocrine induction, we determined that Notch-inhibition with *N*-[*N*-(3,5-difluorophenacetyl)-L-alanyl]-*S*-phenylglycine *t*-butyl ester (DAPT) during aggregation can influence cell-fate determination toward an endocrine/β-cell fate following implantation in mice, increase fasting serum human C-peptide concentrations, and improve glucose clearance.

## Results

### Harvesting of Kit-derived pancreatic progenitor monolayer and subsequent aggregation to generate relatively uniform spheroids

**Figure 1:**
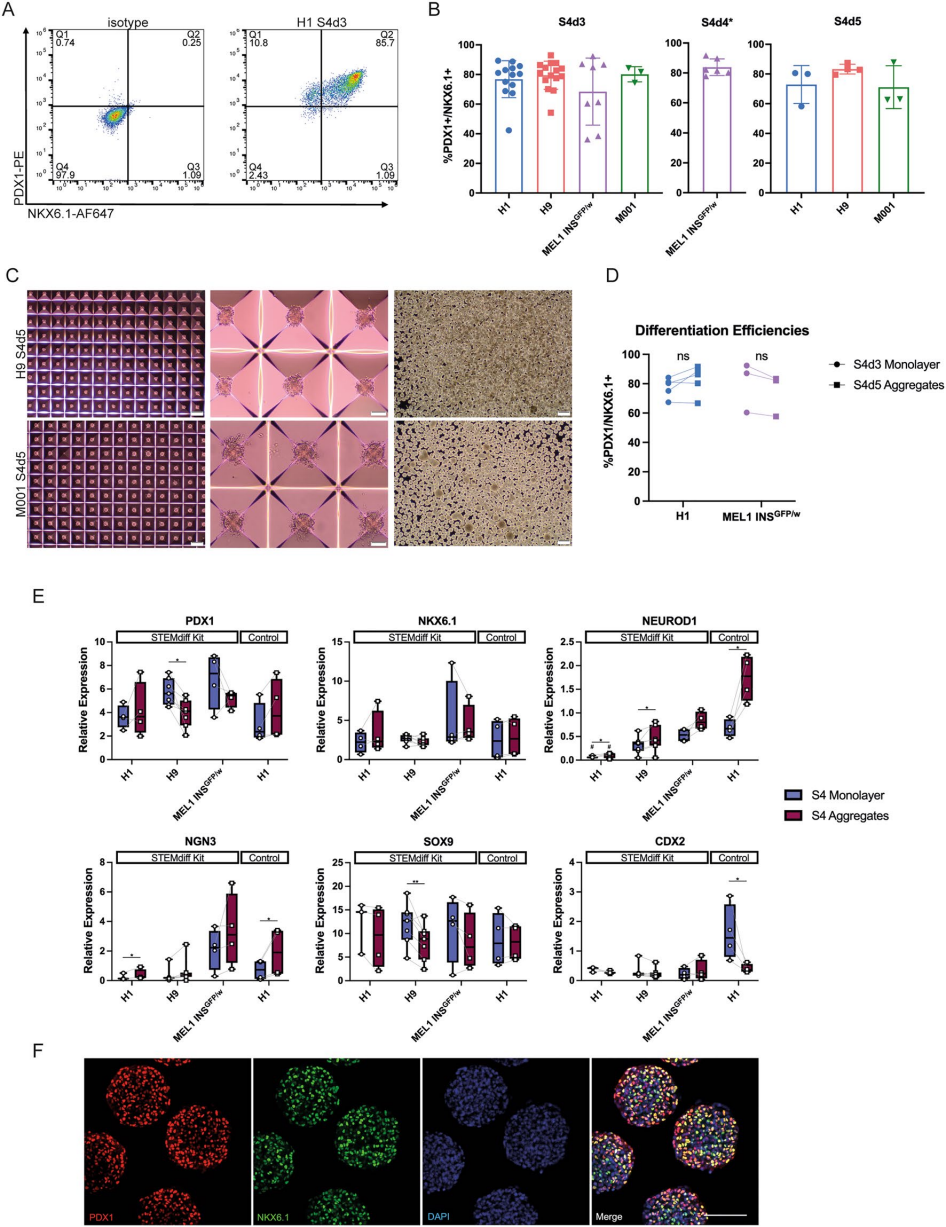
Characterization of STEMDiff™ Pancreatic Progenitor Kit-derived pancreatic progenitors pre- and post-aggregation. Cells aggregated at 750 cells/aggregate in AggreWell™400 plates for 48 h (**C**–**E**) or 3000 cells/aggregate in AggreWell™800 plates for 24 h (**F**). (**A**) Representative flow cytometry analysis for PDX1 and NKX6.1 at stage 4, day 3 of a Kit differentiation in H1 PSCs. (**B**) Combined flow cytometry data of Kit-derived stage 4 pancreatic progenitors from various PSC lines (n = 3–15 independent experiments). Data are presented as mean ± SD. *Note that the S4d4 data are from cells that had been differentiated for 3 days at Stage 1 instead of 2 days. (**C**) Phase contrast images following aggregation. Scale bars of left and right column = 400 µm, scale bars of center column = 100 µm. (**D**) Combined flow cytometry data for PDX1 and NKX6.1 from two different cell lines, comparing pre-aggregation at stage 4, day 3 and post-aggregation at stage 4, day 5 (n = 3–4 independent experiments). A paired t-test was performed, but no significance was found. (**E**) Gene expression analysis comparing S4d3 monolayer and S4d5 aggregated pancreatic progenitors from various cell lines (n = 3–7 independent experiments). The control differentiation is based off an adapted Rezania, 2014 protocol. *PDX1*, *NKX6.1*, and *NEUROD1* are displayed relative to human islet, *NGN3* is displayed relative to a control stage 5 differentiation, *SOX9* is displayed relative to whole human pancreas, and *CDX2* is displayed relative to human small intestine. Note that some data points are shared with Figs. 3E and S5B to facilitate interpretation. *p < 0.05; **p < 0.005 comparing monolayer vs. aggregates by paired t-test. # *p* < 0.05 comparing monolayer/aggregate H1 Kit differentiations vs. monolayer/aggregate H1 control differentiations by unpaired two-tailed t-test with Welch correction. (**F**) Representative whole-mount immunostaining images of Stage 4 aggregated MEL1-INS^GFP/w^ cells. PDX1 (red); NKX6.1 (green); DAPI (blue). Scale bar = 100 μm.

We first sought to differentiate various PSC lines to Stage 4 pancreatic progenitors using the STEMdiff™ Pancreatic Progenitor Kit and form aggregates afterwards using the AggreWell™ workflow. Differentiations were efficient with the PDX1 + /NKX6.1 + cells often comprising > 60% of the population, even when we shortened the length of S4 culture from the recommended 5 days to 3 days (Fig. 1A and B). Time-course analysis of PDX1 and NKX6.1 expression during differentiation in three PSC lines revealed increased NKX6.1 expression after the first 24 h of Stage 4, plateauing by the third day (Fig. S1). This expression was highest in H9 ESCs compared to R038 and 1C iPSCs. GP2, a glycoprotein enriched in pancreatic progenitors^28,29^, increased by the end of Stage 4, Day 2, one day following NKX6.1 upregulation (Fig. S2). In H9 ECS, PDX1 expression was observed in > 90% of the cells by the end of Stage 3 and remained high throughout Stage 4. In both 1C and R038 iPSCs, PDX1 expression was below that observed for the H9 line at the end of Stage 3 but continued to increase during Stage 4 culture (Fig. S1). We observed an average 2.2- to 2.9-fold expansion in cell number during differentiation, depending on the cell line used (Figures S3A and S3B). The viability of the cells remained stable throughout the differentiation at 77.9% ± 9.6%, 76.4% ± 6.1%, and 72.3% ± 11.5% for H9, 1C, and R038 cells respectively (Fig. S3C). While variation in differentiation efficiencies were seen between different cell lines, the overall trends indicated peak efficiencies were reached at the end of the third day of Stage 4.

We reasoned that we would be able to harvest differentiating monolayers at Stage 4, Day 3 and aggregate them for 48 h in the same Stage 4 medium without affecting the differentiation efficiencies. This aligned well with the Kit workflow, as Stage 4 medium can be prepared all at once for the first three days, and then again for the final two days. Using the AggreWell™ system, we were able to aggregate various Stage 4 cell lines into near uniform clusters, without significantly altering the % PDX1 + /NKX6.1 + population (Fig. 1C and D). RNA expression analysis of Kit-derived cells between the harvested monolayer and aggregates did not reveal any transcriptional changes to the *PDX1*, *NKX6.1*, or *SOX9* genes other than small, but significant decreases in *PDX1* and *SOX9* expression in H9 ESCs (Fig. 1E). Expression of *CDX2*, a marker for off-target intestinal differentiation, remained low in Kit-differentiations. Using a control protocol (modified Rezania, 2014 differentiation), we observed relatively high expression levels of CDX2 in H1 cells at Stage 4, day 3, which significantly decreased during the aggregation step (monolayer 1.61 ± 0.94; aggregates 0.41 ± 0.15; *p* = 0.020) (Fig. 1E). During aggregation of Kit generated cells, small but significant increases were observed in the levels of *NGN3* in H1 cells (monolayer 0.25 ± 0.23; aggregates 0.46 ± 0.40; *p* = 0.038) and small but significant increases in *NEUROD1* expression were observed in H1 cells (monolayer 0.07 ± 0.03; aggregates 0.13 ± 0.02; *p* = 0.045) and H9 cells (monolayer 0.32 ± 0.18; aggregates 0.39 ± 0.25; *p* = 0.030) (Fig. 1E). H1 cells aggregated following Kit differentiations had significantly lower expression of *NEUROD1* compared to H1 cells differentiated in the control condition (Kit aggregates 0.11 ± 0.06; control aggregates 1.74 ± 0.49; *p* = 0.006), though the expression of *NEUROD1* was also significantly lower pre-aggregation (Kit monolayer 0.07 ± 0.03; control monolayer 0.68 ± 0.19; *p* = 0.006) (Fig. 1E). Aggregates were strained through a 37 μm reversible filter to remove any non-aggregated cells, which had a lower viability compared to the intact aggregates (Fig. S3D). Immunostaining of aggregated MEL1-INS^GFP/w^ pancreatic progenitors revealed many cells immunoreactive for both PDX1 and NKX6.1 (Fig. 1F). Thus, the early harvest of the Kit-derived pancreatic progenitor monolayer and subsequent aggregation can be used to generate pancreatic progenitor spheroids ready for transition into suspension culture.

### Generation of functional islet-like clusters from the Kit-derived pancreatic progenitors

**Figure 2:**
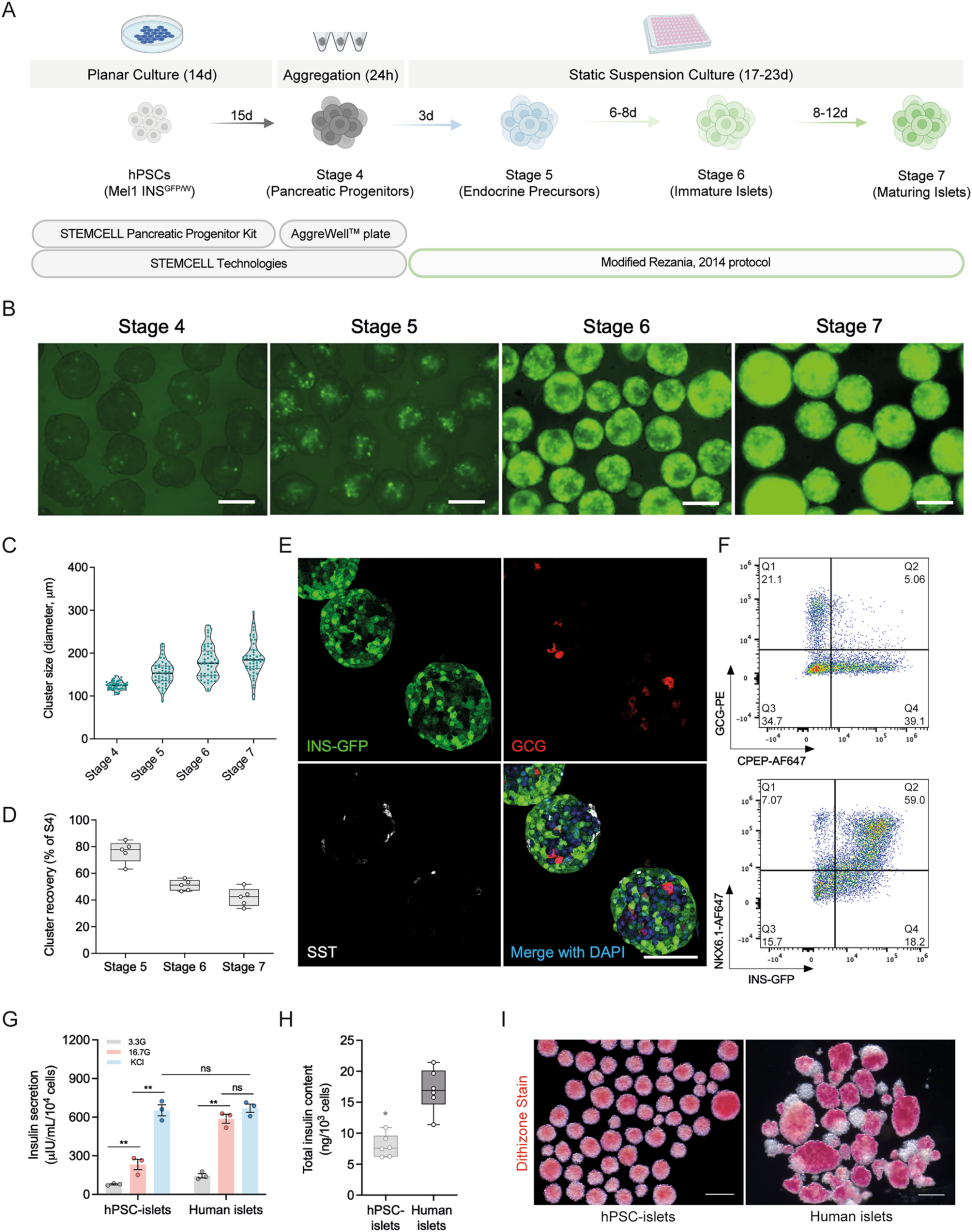
Generation of functional islet-like clusters from Kit-derived pancreatic progenitors. (**A**) Schematic diagram for directed differentiation of human pluripotent stem cells to insulin-secreting islet-like clusters. The STEMdiff™ Pancreatic Progenitor Kit was used for the induction of pancreatic progenitor cells, AggreWell™800 microwell plates were used for aggregation of cells into pancreatic progenitor clusters at 3000 cells/cluster, and a modified Rezania, 2014 protocol was used for endocrine induction during the last three stages. (**B**) Representative images show gradual induction of insulin-expressing cells in live cell cultures with the insulin reporter Mel1-INS^GFP/w^ hESC line. Scale bars = 100 μm. (**C**) Quantification of cluster size at indicated stages (n = 3 independent experiments). (**D**) Quantification of cluster recovery rate at indicated stages (n = 5 independent experiments). (**E**) Representative whole-mount immunostaining images showing that all the three major islet cell types were induced in Stage 7 hPSC-islets. Insulin is indicated by INS^GFP^ signal (green); glucagon, GCG (red); somatostatin, SST (gray); DAPI (blue). Scale bar, 100 μm. (**F**) Representative flow cytometry analysis for C-peptide (CPEP), glucagon (GCG), INS-GFP, and NKX6.1 in Stage 7 hPSC-islets. (**G**) Static GSIS assays showing insulin secretion from Stage 7 hPSC-islets and primary human islets (n = 3 independent experiments) in response to low glucose (3.3G, 3.3 mM glucose), high glucose (16.7G, 16.7 mM glucose), and 30 mM KCl depolarization challenge. Data are presented as mean ± SEM. **p < 0.005 by unpaired two-tailed t-test. (**H**) Total insulin content of Stage 7 hPSC-islets and primary human islets (n = 3 independent experiments with two technical replicates each). *p < 0.05 versus human islets by unpaired two-tailed t-test. (**I**) Representative images showing dithizone-stained Stage 7 hPSC-islets and a typical human islet preparation. Scale bar = 200 μm.

To examine the capacity of the Kit-derived pancreatic progenitors to commit to endocrine lineages, we further differentiated the spheroids following our previously reported protocol^18^ with small modifications in a static 96-well plate suspension culture format (Fig. 2A). With the use of the MEL1-INS^GFP/w^ hESC insulin reporter line^30^, we were able to visualize the differentiation by monitoring the fluorescence expression pattern. First, we observed that insulin, as indicated by GFP, was induced as early as Stage 4, though by flow cytometry analysis only 3%-5% of Stage 4 cells expressed insulin (data not shown). From Stage 5 onward, we observed more cells becoming insulin positive as indicated by increasing area and intensity of GFP fluorescence within the clusters during differentiation (Fig. 2B). Stage 4 aggregates were relatively uniform, with an average cluster size of 122 ± 7 μm, which gradually increased to 195 ± 24 μm by Stage 7 (Fig. 2C), suggesting some cell division and/or cluster fusion. The cluster yield (the number of clusters retrieved at the end of each indicated stage divided by the initial number of Stage 4 cluster input) remained as high as 80% at the end of Stage 5 but decreased to about 45% by Stage 7 (Fig. 2D). While aggregate fusion could contribute to this decrease, entire clusters were also observed shrinking and disappearing over time (data not shown).

We next characterized the cell composition of the differentiated islet-like clusters at Stage 7 and found that chromogranin A (CHGA) and NEUROD1 immunoreactivity was present in 80–95% of cells, suggesting a majority composition of endocrine cell types (Fig. S4A). PDX1 immunoreactivity was observed in > 95% of Stage 7 cells indicating that cells within the aggregates had at the very least differentiated to a posterior foregut cell type (Fig. S4B). All three major islet cell types, including insulin-expressing β-cells, glucagon-expressing ɑ-cells, and somatostatin-expressing δ-cells were present in our spheroids, with ~ 5% bi-hormonal cells co-expressing C-peptide and glucagon in the total cell population (Figs. 2E,F, and S4A). A further 5–10% of the aggregates were comprised of pancreatic polypeptide-expressing PP cells and SLC18A1-expressing enterochromaffin cells (Fig. S4A and S4C). In addition, we were able to generate 50–60% insulin + /NKX6.1 + cells in our Stage 7 clusters (Fig. 2F), indicating an efficient induction of β-cells. Notably, C-peptide immunoreactivity was lower than the GFP + population (Fig. 2F) suggesting differences in post-transcriptional/post-translational control between the two genes or that some cells had activated the insulin gene, later shut it off, and GFP levels were decreasing but still detectable. In static glucose-stimulated insulin secretion (GSIS) assays, Stage 7 aggregates secreted significantly more insulin in response to high glucose compared to basal and displayed comparable insulin secretion capacity under depolarization challenge to human islets (Fig. 2G). The insulin secretion fold-change between low and high glucose conditions was not significant between our hPSC-islets and human islet controls, though the hPSC-islets did have a significantly greater fold secretion in the presence of KCl associated with their lower basal secretion levels (Fig. S4D). Although the total insulin content of our maturing islet-like clusters was about half the amount of human islets (Fig. 2H), our stem cell-derived Stage 7 clusters displayed more uniform insulin granule distribution between different clusters relative to human islets as indicated by dithizone staining (Fig. 2I and S4E). Collectively, these data suggest that the Kit-derived pancreatic progenitors can efficiently differentiate into functional islet-like clusters in vitro.

### FGF7 improves aggregate survival following transition into suspension culture

**Figure 3:**
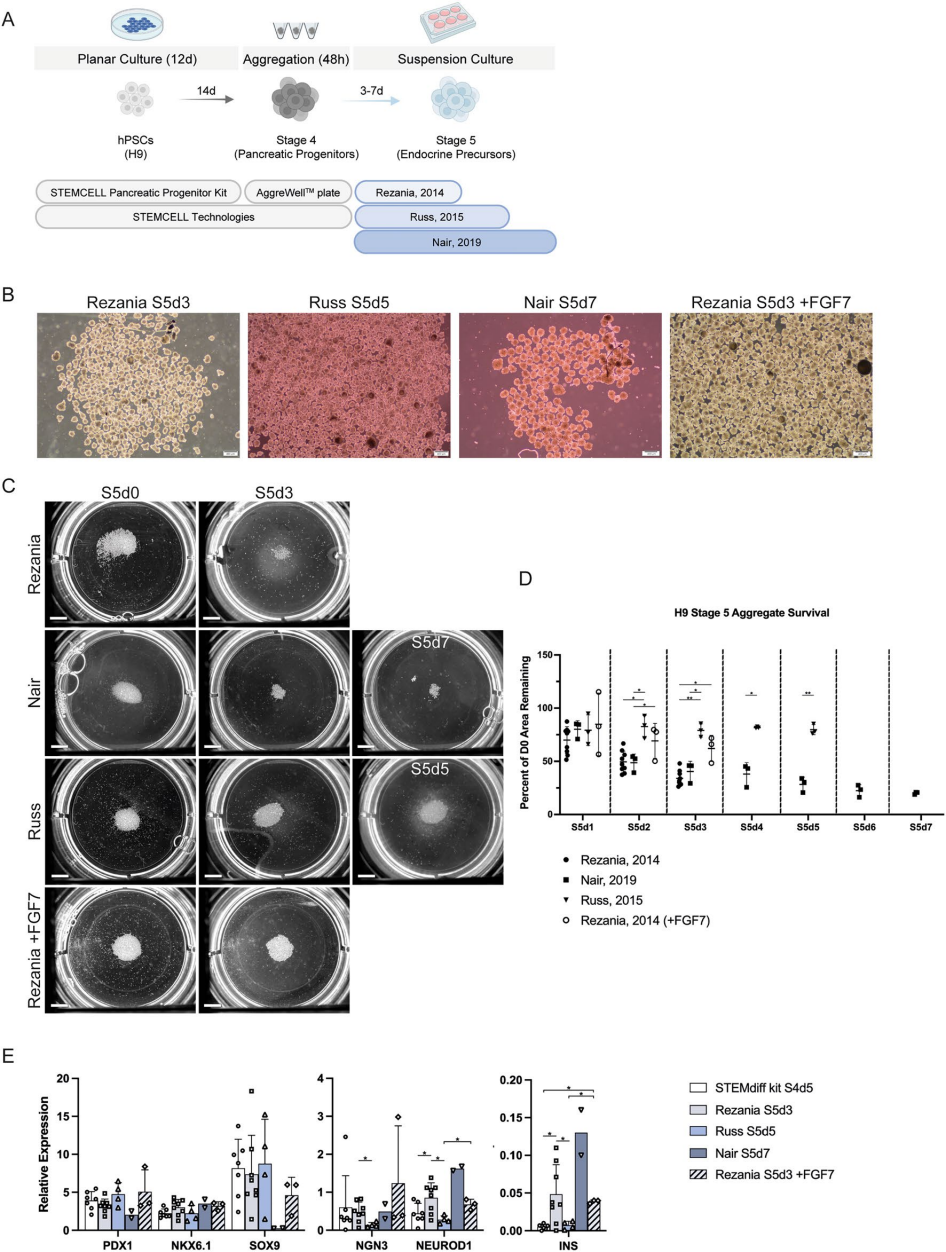
Comparing various stage 5 culture conditions using H9-derived cells. (**A**) Three stage 5 endocrine precursor medium formulations were tested for how well they work with the STEMdiff™ Pancreatic Progenitor Kit following aggregation at 750 cells/cluster in AggreWell™400 plates and transition into shaker suspension culture in a 6-well plate format. (**B**) Phase contrast images of the aggregates at the end of various stage 5 culture conditions. Scale bar = 400 µm. (**C**) Whole-well imaging of suspension culture wells from different stage 5 culture conditions. Scale bar = 50 mm. (**D**) Quantification of aggregate area every day during S5 culture relative to the initial seed (n = 3–9 independent experiments). Data are presented as mean ± SD. *p < 0.05, **p < 0.005, ***p < 0.0005 by unpaired two-tailed t-test with Welch correction. (**E**) Gene expression analysis of aggregates cultured in the stage 5 conditions (n = 2–8 independent experiments). *PDX1*, *NKX6.1*, *NEUROD1*, and *INS* are displayed relative to human islet, *SOX9* is displayed relative to whole human pancreas, and *NGN3* is displayed relative to a control stage 5 differentiation. Note that some data points are shared with Figs. 1E and S5B to facilitate interpretation. Data are presented as mean ± SD. *p < 0.05 by unpaired two-tailed t-test with Welch correction.

In recognition that our 96-well static suspension culture system is not suited for scaled-up production, we next attempted to transition pancreatic progenitor spheroids to 6-well shaker-plate suspension culture, testing three previously reported Stage 5 formulations on H9 PSCs^18,20,22^ (Fig. 3A, Supplemental Table 1). Cluster sizes were similar between the different tested media, though there were clear differences in aggregate numbers (Fig. 3B). Using a whole-well imaging setup, we visualized the total aggregate area over the course of the culture (Figs. 3C and S5A). Using a CellProfiler script, we quantified the area of the plate taken up by aggregates and monitored changes over time. This macro was able to identify aggregates that were evenly distributed in the well or that had been manually swirled to the center of the well (Fig. S5A). Comparing the same wells with an even or central aggregate distribution, the macro was able to identify comparable total aggregate area (Fig. S5B). Some aggregates were localized along the well edges and out of the macro detection area in wells with an even spread. This likely resulted in the small difference observed when comparing the total aggregate areas between the two distributions and we therefore chose to continue imaging wells following a gentle swirl to bring the aggregates towards the center.

Our initial trials compared the Rezania, 2014 and Russ, 2015 Stage 5 formulations. Cell losses were considerably greater using the Rezania protocol, with only 34.9 ± 8.1% of the initial aggregate area remaining following 3 days in culture, as compared to 79.8% ± 4.7% of the initial aggregate area remaining in the Russ condition following 5 days of culture (Figs. 3D and S6A). Interestingly, the aggregates cultured with the Rezania formulation had increased gene expression of *NGN3, NEUROD1,* and *INS* when compared to the Russ, 2015 formulation (Fig. 3E). We next set out to compare the Rezania, 2014 and Nair, 2019 Stage 5 formulations. It was apparent that the cell losses were similar between the two, with the Nair condition retaining only 40.4% ± 9.6% of the initial aggregate area remaining following 3 days in culture, and 20.1% ± 1.2% at the end of 7 days in culture (Figs. 3D and S6A). Compared to the Stage 4 aggregates, the gene expression analysis of cells cultured with the Nair formulation showed increases in *NGN3, NEUROD1,* and *INS*, similar to the Rezania condition (Fig. 3E). Comparing endocrine progenitor gene expression from the H9 Rezania Stage 5 runs to Stage 5 cells from a control protocol in H1 cells (modified Rezania, 2014), it was apparent that *NEUROD1* levels were lower in the H9 condition, though *NGN3* and *INS* expression levels were not significantly different (Fig. S6B). *NEUROD1* levels were also lower in Rezania Stage 4 runs compared to Stage 4 cells from the control protocol, but *INS* expression was higher. Perhaps the variation in gene expression can be attributed to inherent differences between the H1 and H9 ESCs used in the control and sample groups, respectively, or it could indicate that further protocol optimization is warranted to match the gene expression pattern of the control protocol.

When comparing the formulations of the three tested Stage 5 media, we noticed that FGF7 was present in media used in the Russ protocol but was absent in the media of the other two protocols. We ran several trials, either adding FGF7 to the Rezania medium, or removing it from the Russ medium. The addition of FGF7 to the Rezania medium increased aggregate survival up to 63% ± 10.2% following 3 days in culture without significantly affecting the expression of genes analyzed (Figs. 3D,E, and fig. S6A). When FGF7 was removed from the Russ medium, aggregate survival decreased compared to the original formulation, but was still higher than the Rezania condition (Fig. S6A).

In one trial, we added twice as many aggregates into the suspension culture to test whether initial aggregate mass affects the rate of cell loss. While there were more aggregates present after 3 days of culture in the Stage 5 Rezania medium, the rate of loss was similar to the control (Fig. S6A). Several other trials were performed with the H1 and M001 hPSC lines, further supporting the aggregate loss observations in H9 cultures. Kit-derived H1 and M001 pancreatic progenitors further differentiated with the Stage 5 Rezania formulation had an increased cell mass when treated with FGF7, though not as striking as with the H9 cells (Fig. S6A). M001 aggregates in the Stage 5 Russ formulation had considerably more aggregate mass following 5 days of culture compared to the Rezania and Nair conditions (Fig. S6A). We conclude that FGF7 positively affects aggregate survival in H9 Stage 5 culture, though its absence may not fully account for the observed cell losses in the Rezania cultures and more work is needed to establish its effects in other cell lines.

### Notch-inhibition during pancreatic progenitor aggregation increases endocrine gene expression and decreases SOX9 expression

An interesting observation that came from the Stage 5 protocol testing was the downregulation of *SOX9* expression with the Nair formulation compared to the Rezania and Russ formulations (Fig. 3E). Our control differentiation protocol was also able to downregulate *SOX9* expression by the end of Stage 5 (Fig. S6B). The STEMDiff™ Pancreatic Progenitor Kit generates multipotent pancreatic progenitors that should express *SOX9*, but continued expression would suppress endocrine commitment during later stages and bias the cells towards a ductal fate. We hypothesized that the presence of the Notch inhibitor XXi in the Nair medium was responsible for downregulating *SOX9* expression and upregulating *NEUROD1* expression. Since high % PDX1 + /NKX6.1 + efficiencies were present in Kit-differentiations prior to aggregation, we tested Notch inhibition during aggregation to downregulate *SOX9* expression and induce endocrine gene expression.

**Figure 4:**
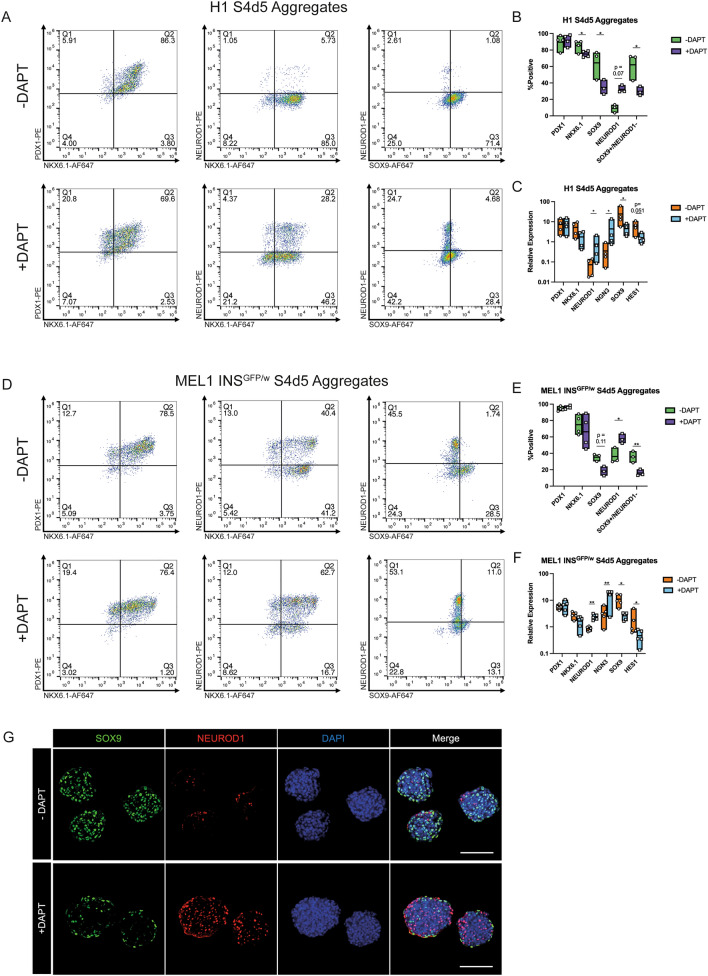
Analysis of H1 and MEL1-INS^GFP/w^ lines following stage 4 aggregation with or without 10 μM DAPT for 48 h. Cells aggregated at 750 cells/cluster in AggreWell™400 plates (**A**–**F**) or AggreWell™800 plates (**G**). (**A**, **D**) Representative flow cytometry analysis for PDX1, NKX6.1, NEUROD1, and SOX9. (**B**, **E**) Combined flow cytometry data (n = 3–4 independent experiments). (**C**, **F**) Gene expression analysis (n = 3–4 independent experiments). *PDX1*, *NKX6.1*, *NEUROD1*, and *HES1* are displayed relative to human islet, *SOX9* is displayed relative to whole human pancreas, and *NGN3* is displayed relative to a control stage 5 differentiation. (**G**) Representative whole-mount immunostaining images of Stage 4 MEL1-INS^GFP/w^ aggregates. SOX9 (green); NEUROD1 (red); DAPI (blue). Scale bar = 100 μm. *p < 0.05, **p < 0.005 by paired t-test.

Using the gamma-secretase/Notch inhibitor DAPT during the 48 h aggregation step did not greatly change the morphology of generated clusters, and aggregate size was not significantly altered (Figures S7A, S7B, and S7C). With DAPT treatment, we observed a significant increase in the % NEUROD1 + cell population of the MEL1-INS^GFP/w^ cells and an increase trending toward significance in the % NEUROD1 + population of the H1 cells (Figs. 4A,B,D,E, and S7D). In the H1 experiments, the % SOX9 cell population decreased with Notch inhibition and the % NEUROD1 + /SOX9 + was consistently low (< 5%), confirming that these two markers are rarely co-expressed (Fig. 4A and B). While the % SOX9 + population did not significantly change in the MEL1-INS^GFP/w^ cells by flow analysis (Fig. 4E), it trended downward and the % SOX9 + /NEUROD1- population did significantly decrease (Fig. 4D). PDX1 and NKX6.1 immunoreactive populations were comparable between the two treatment groups in both cell lines, though there was a significant decrease in NKX6.1 immunoreactivity with DAPT treatment in the H1 experiments (Figs. 4B,E, S7D, and S7E). A decrease in the NKX6.1 population could indicate a transition of a subset of cells towards an α-cell fate. Interestingly, the MEL1-INS^GFP/w^ aggregates had a consistently higher % NEUROD1 + population compared to H1 cells, even in the untreated group (Fig. 4E), suggesting that these cells are more primed for endocrine commitment at the end of the pancreatic progenitor stage when using the STEMdiff™ Kit. The immunoreactivity for CHGA, another endocrine cell marker, also increased with DAPT treatment (Fig. S7D and S7F).

At the gene expression level, the endocrine transcription factors *NEUROD1* and *NGN3* significantly increased with Notch inhibition in both cell lines (Fig. 4C and F). *SOX9* expression significantly decreased in the presence of DAPT (Fig. 4C and F), as did *HES1* expression in the MEL1-INS^GFP/w^ cell line (Fig. 4F). *HES1* expression appeared to decrease in H1 cells as well, but this difference was not significant. In both cell lines, *PDX1* and *NKX6.1* expression did not change between the treatment groups (Fig. 4C and F). Whole-mount immunostaining of MEL1-INS^GFP/w^ spheroids confirmed the qPCR results, with a substantial increase in NEUROD1 and decrease in SOX9 immunoreactivities in response to DAPT treatment (Fig. 4G and D). Interestingly, these results match the H1 flow cytometry data, but not that for the MEL1-INS^GFP/w^ cells (Fig. 4B and E). It is likely that the flow cytometry staining of DAPT-treated MEL1-INS^GFP/w^ aggregates was inaccurate, reporting a low-expressing false-positive SOX9 stain, but we opted not to adjust our gating away from the isotype control. We conclude that endocrine commitment can be initiated during aggregation through Notch inhibition in Stage 4 medium.

### Notch-inhibition of pancreatic progenitors prior to implantation increases endocrine fate within the graft

**Figure 5:**
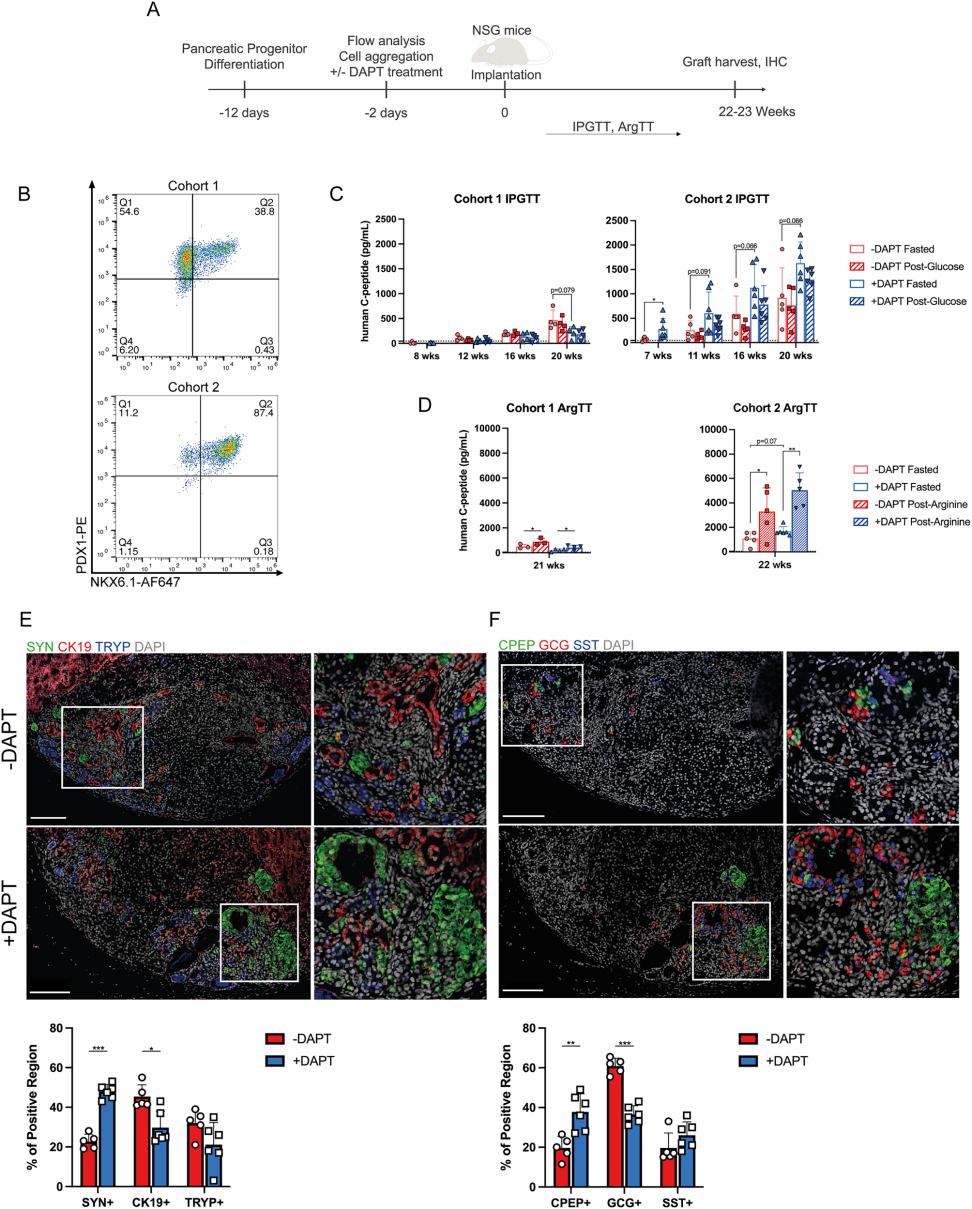
Notch-inhibition of pancreatic progenitors prior to implantation improves graft outcomes. (**A**) Experimental timeline. Following differentiation with the STEMdiff™ Pancreatic Progenitor Kit, MEL1-INS^GFP/w^ cells were aggregated for 48 h with or without DAPT treatment prior to implantation under the kidney capsule of male NSG mice. Over the following 22 weeks, the mice were challenged with 1 g/kg IP glucose or 2 g/kg IP arginine injections. At the end of the experiment, grafts were explanted for histological analysis. (**B**) Two independent differentiations and surgeries comprise cohorts 1 and 2. The differentiation efficiency of cells going into cohort 1 was lower than those going into cohort 2. (**C**) Fasting and 15-min post-glucose plasma human C-peptide levels measured from mice in both cohorts 7–20 weeks post-implant during the course of the experiment. Note that one mouse in the + DAPT group of cohort 2 died from an unknown cause at week 15. (**D**) Fasting and 15-min post-arginine plasma human C-peptide levels measured from mice in both cohorts 21–22 weeks post-implant. Note that one mouse in the -DAPT group of cohort 1 was too sick to undergo the arginine tolerance test. (**E**, **F**) Immunohistochemical staining of the explanted grafts of cohort 2. Stains were for synaptophysin (SYN), CK19, and trypsin (TRYP) or C-peptide (CPEP), glucagon (GCG), and somatostatin (SST). A representative graft from each treatment group is shown as well as the summary data from all mice. Scale bar = 500 μm. n = 4–7 mice per treatment group per cohort. Bar data are presented as mean ± SD *p < 0.05; **p < 0.005; ***p < 0.0005 by unpaired two-tailed t-test with Welsh correction between treatment groups or paired t-test within same treatment group.

Next, we explored whether DAPT treatment of pancreatic progenitor aggregates can improve in vivo graft outcomes following implantation in normoglycemic mice (Fig. 5A). Two separate experimental cohorts were tested, corresponding to two independent differentiation runs of the MEL1-INS^GFP/w^ cell line. The first round of pancreatic progenitor differentiations going into cohort 1 had a lower % PDX1 + /NKX6.1 + population compared to the second round going into cohort 2 (Fig. 5B), though the % PDX1 + population was still > 90%. We performed the implantation study in cohort 1 to determine if Notch inhibition prior to NKX6.1 induction would lead to an increased proportion of glucagon-expressing cells following graft maturation. Following aggregation with or without DAPT, spheroids were collected and implanted under the kidney capsule of male NSG mice. Cohort 1 mice received 3.2 × 10^6^ cells, while cohort 2 mice received 4.9 × 10^6^ cells per animal.

The overall graft performance of cohort 2 was better than cohort 1, as basal and post-glucose human C-peptide levels were higher (Fig. 5C and D). Notch inhibition did not appear to influence glucose clearance in cohort 1 during IPGTT testing, while at 20 weeks post implant in cohort 2 there was a significant decrease in blood glucose AUC (Figures S8A and S8B). Similarly, serum human C-peptide levels in cohort 1 did not differ between treatment groups at any time point monitored, but in cohort 2 fasted human C-peptide levels were significantly higher in the DAPT group 7-weeks post-implant (Fig. 5C). We did not observe glucose-stimulated human C-peptide secretion at any time point during IPGTT testing (Figs. 5C and S8C), though human C-peptide levels were significantly higher in the DAPT treated group of cohort 2 at certain IPGTT timepoints (Fig. S8C). In the non-diabetic animals, mouse C-peptide did increase with glucose challenge, as expected (Fig. S8D). Interestingly, 15 min post-glucose challenge there was significantly less mouse C-peptide in the serum of mice within the DAPT treated group of cohort 2, suggesting a greater contribution to glucose homeostasis from the implanted cells. An arginine challenge induced human C-peptide release (Figs. 5D and S8F). As stimulation by arginine is independent of glucose metabolism and K_ATP_-channel closing^31^, the observed secretion from arginine and lack of stimulation from glucose suggests the grafts had not yet fully matured. Of note, during the arginine tolerance test one mouse in the + DAPT group of cohort 1 and three mice in cohort 2 had to be rescued from hypoglycemia 30-min post-injection (Fig. S8E). These mice had to be omitted but the data suggests that a stronger β-cell response to arginine was present in mice that received DAPT-treated cells. Of note, too much insulin release leading to hypoglycemia is not desirable.

At 22–23 weeks post-implant, kidneys were collected from the mice and fixed for further analysis. Immunohistochemical examination of the cohort 1 grafts did not reveal any differences in the distribution of endocrine, ductal, or exocrine cells between the two treatment groups (Fig. S9A). C-peptide, glucagon, and somatostatin immunoreactivity within the endocrine compartment also remained similar between the two groups (Fig. S9B). These data and the previous functional data highlight the importance of implanting a high PDX1 + /NKX6.1 + pancreatic progenitor population. Within the cohort 2 grafts, DAPT treatment increased the synaptophysin immunoreactive areas and decreased CK19 immunoreactive areas, suggesting an increase in the prevalence of endocrine cells at the expense of ductal cells (Fig. 5E). Within the endocrine compartment, DAPT treatment increased the C-peptide immunoreactive areas and decreased the glucagon immunoreactivity of the grafts in cohort 2 (Fig. 5F). We conclude that Notch-inhibition during pancreatic progenitor aggregation can improve fasting human C-peptide concentrations, glucose clearance, and cell-fate determination following implantation into mice.

## Discussion

Here, we have developed a protocol to harvest pancreatic endoderm derived from STEMdiff™ Pancreatic Progenitor Kit differentiations, aggregate them into relatively uniform spheroids, and transition them into static or shaker suspension culture. Our initial work to monitor the expression of PDX1, NKX6.1, and GP2 over time revealed an increase in the % PDX1 + population by the end of Stage 3 and an increase in the % NKX6.1 + population within the first two days of Stage 4 differentiation. In the cell lines we tested, the maximum % PDX1 + /NKX6.1 + population was reached and plateaued 2- 3 days into Stage 4. In contrast to a previous report of GP2 expression describing its activation prior to NKX6.1^28^, we observed GP2 increasing 24 h after NKX6.1 upregulation. Our conflicting results suggest that GP2 expression dynamics in relation to NKX6.1 could be protocol specific or the previously reported expression dynamics in the NKX6.1^GFP/w^ cell line was affected by the functional loss of one NKX6.1 allele, a possibility since NKX6.1 can bind to its own enhancer element to maintain its expression^32^.

Aggregation using the AggreWell™ platform generated relatively uniform clusters of pancreatic progenitors, further demonstrating the utility of Stage 4 spheroid generation with microwell technology^24,33^. Using static suspension culture in 96-well plates, we generated functional islet-like cells that could release insulin in response to a glucose challenge. By using the MEL1-INS^GFP/w^ hPSC line^30^, we were able to track clusters in individual 96-wells and monitor differentiation through their GFP fluorescence. By characterizing the endocrine cell composition of Stage 7 aggregates, we demonstrated that our protocol produces islet-like clusters composed of the major islet cell types. Notably, the fraction of insulin + /glucagon + bi-hormonal cells remains low (~ 5%), and ~ 60% insulin + /NKX6.1 + cells are present within the Stage 7 cell population, suggesting an efficient induction of β-cells. Considering the Mel1-INS^GFP/w^ hESC line contains only one functional insulin allele, it is promising to see that the total insulin content of hPSC-islets reaches approximately half the amount found in our human islet preparations. The even distribution of insulin expression in relatively uniform-sized hPSC-islets makes them suitable for further experimentation. Our static 96-well culture protocol enables scaled-out screening of different culture conditions but may not be practical for scaled-up production. Considering that the glucose-stimulated insulin secretion of our hPSC-islets is still lower than primary human islets, future efforts of protocol optimization are needed to improve their functionality.

Shaker suspension culture was used at a larger scale to compare various endocrine media formulations and their impact on aggregate survival and gene expression. Though we observed considerable differences in aggregate survival rate, the remaining spheroids were similar in size and shape between different culture conditions (Fig. 3B). This indicates that cells were not dying off all clusters evenly, but that certain aggregates had a survival advantage over others. Additionally, all conditions tested were run in the same medium volume and at the same orbital shaker speed, making shear forces and hypoxia unlikely major contributors to aggregate loss at Stage 5. Research has linked the repression of Notch signaling to increased apoptosis in adult human and mouse islets in a dose-dependent manner^34^. The same study showed that removal of serum or the pharmacological inhibition of growth factor signaling pathways during human and mouse islet culture resulted in a reduction in activated Notch protein. As Notch inhibition is important to transition pancreatic progenitors into endocrine tissue, it is possible that some cells in suspension culture inappropriately reached an apoptotic threshold that caused total spheroid destruction. While an exogenous source of Notch inhibition was provided in the Nair Stage 5 formulation, it was not present in the Rezania Stage 5 medium. The lack of growth factors in the Stage 5 medium formulations of both Rezania and Nair protocols also explain the increased aggregate loss, as FGF7 supplementation improved survival. In contrast, the 96-well differentiation cultures using the Rezania formulations had less cell loss during Stage 5, but a large loss through Stage 6 culture (Fig. 2D) coinciding with the addition of the Notch inhibitor GSi XX. It is possible that there are greater stresses from the transfer of aggregates into shaker culture than transfer into the static system, which could explain the discrepancies in aggregate survival between the two formats.

While we report differences in aggregate survival and gene expression in the various Stage 5 media formulations tested, we do not discount the validity of the respective protocols. As previously reported, there is variation in Stage 4 populations generated using different protocols^35^ and downstream media formulations may be optimized for each specific population. Importantly, both the Russ and Nair differentiation protocols use suspension culture differentiation to generate pancreatic progenitors^20,22^. Pairing their respective Stage 5 formulations with recently harvested and aggregated Kit-derived Stage 4 cells may give different outcomes than originally reported. When paired with the STEMdiff™ Pancreatic Progenitor Kit, the Rezania and Nair Stage 5 formulations had increased endocrine gene expression compared to the Russ Stage 5 formulation, but the latter did not cause aggregate loss to the same extent. Because FGF7 was unique to the Russ formulation, we supplemented it in the Rezania media and improved survival while retaining endocrine gene expression. During islet development, pancreatic progenitors exit the cell cycle following the induction of *NGN3*, which is correlated with cell cycle lengthening and the upregulation of the cell cycle inhibitor, *CDKN1A*^36,37^. The addition of FGF7 during Stage 5 may have had a negative impact on proper endocrine commitment that was not detected by our gene expression studies. Further experimentation is needed to determine the impact of Stage 5 growth factor treatment on β-cell differentiation efficiencies, including its use in combination with Notch inhibition.

Whereas the Pancreatic Progenitor Kit generates true multipotent Stage 4 cells that can continue to differentiate into exocrine, ductal, and endocrine tissues, β-cell differentiation protocols may already have biased cells to commit to the endocrine pathway by this stage. It could be for this reason that *SOX9* expression remained high following Stage 5 differentiation in the Rezania medium (Fig. S6B) indicating that we needed to consider further supplementation to reduce its expression. Using DAPT to inhibit Notch signaling during aggregation, we were able to drive down SOX9 levels and upregulate endocrine genes, consistent with mouse studies^8,38,39^ and a recent study in differentiating PSCs^40^. Interestingly, MEL1-INS^GFP/w^ Stage 4 cells seemed to be more committed to an endocrine fate compared to H1 Stage 4 cells, as a NEUROD1 + population was already emerging (Fig. 4D and E) and *NGN3* and *NEUROD1* gene expression was greater in the reporter line (Fig. 1E) even in the absence of DAPT.

In human β-cell development, *NGN3* expression follows the induction of *NKX6.1*^5^. In mice, earlier *NGN3* upregulation preceding *NKX6.1* activation in a subpopulation of developing cells initiates a primary transition of endocrine differentiation and results in a population resembling ɑ-cells^41,42^. A secondary transition of endocrine differentiation follows, where *NGN3* activation occurs in an NKX6.1 + population^41^. While a primary transition-like step is not thought to occur naturally in human development, early *NGN3* activation during in vitro differentiation of human cells has also been shown to generate cells with an ɑ-like phenotype^43,44^. Because most of the differentiations in our tested cell lines had a high % PDX1 + /NKX6.1 + population by Stage 4, Day 3, we did not think Notch inhibition during aggregation would lead to premature induction of *NGN3* and bias the cells to an ɑ-cell fate. Cells implanted into the cohort 1 mice, however had a lower than average % NKX6.1 + population, but DAPT treatment did not lead to any significant differences in ɑ- or β-cell ratios in the graft (Fig. S9B). While Notch inhibition during aggregation reduced *SOX9* expression and improved endocrine induction by the end of Stage 4 in Kit-derived cells, the impact on further in vitro differentiation remains to be seen. Cells that are further endocrine committed may respond differently to later stage media and optimization would be warranted if the differentiation efficiency to islet-like cells was low. Nevertheless, treating Kit-derived Stage 4 MEL1-INS^GFP/w^ cells with DAPT during aggregation prior to implantation resulted in increased fasting human C-peptide levels and improved glucose tolerance, even though the cell line used only has one functional insulin allele^30^. While we did not observe an increase in serum levels of human C-peptide post-glucose administration, it is possible that a peak occurred prior to our earliest measurement 15-min following glucose injection. Of note, our implantation study was performed in non-diabetic male mice and further studies will be required to determine the effects of Notch inhibition in diabetic and female recipients, as these variables have been found to impact graft maturation and function^45–48^. Grafts from DAPT-treated Stage 4 aggregates also contained higher ratios of endocrine cells to ductal cells and β-cells to ɑ-cells at the end of the experiment. These differences were only observed when using cells that had differentiated efficiently to Stage 4, as the cohort 1 implantation experiment did not show significant functional or cell ratio differences. Improving Stage 4 implantation outcomes is notable, as relying on continued in vitro culture to later stages prior to implantation could reduce cell yields, increase costs, and limit experimentation. The STEMdiff™ Pancreatic Progenitor Kit is also user friendly and optimized for multiple PSCs; improving graft outcomes without first transitioning to more complicated protocols would be ideal.

## Methods

### Cell sources

Human islets for research were provided by the Alberta Diabetes Institute IsletCore at the University of Alberta in Edmonton (www.bcell.org/adi-isletcore) with the assistance of the Human Organ Procurement and Exchange (HOPE) program, Trillium Gift of Life Network (TGLN), and other Canadian organ procurement organizations. Islet isolation was approved by the Human Research Ethics Board at the University of Alberta (Pro00013094). All donors’ families gave informed consent for the use of pancreatic tissue in research.

The H1 and H9 hESC lines were obtained from WiCell Research Institute, Inc. (Madison, WI). The MEL1-INS^GFP/w^ hESC line was obtained from Dr. Edouard G. Stanley (Murdoch Children’s Research Institute and Monash University, Australia) and transitioned off mouse embryonic fibroblasts (MEFs) to Matrigel®. The STiPS-M001 (SCTi002-A) and STiPS-R038 iPSC lines were generated at STEMCELL Technologies (Vancouver, BC). The WLA-1C iPSC line was obtained from the Bill Stanford lab (The Ottawa Hospital Research Institute).

All cell lines have undergone G-banding karyotype analyses with a minimum of 20 cells analyzed by WiCell Research Institute (Madison, WI), confirmed to be mycoplasma-free, and underwent pluripotency verification using the STEMDiff™ Trilineage Differentiation Kit (STEMCELL, Cat# 05230). All experiments with cell lines were approved by the Canadian Stem Cell Oversight Committee and/or UBC Clinical Research Ethics Board. All work with human cells and tissues was conducted in accordance with the 2018 Canadian Tri-Council Policy Statement: Ethical Conduct for Research Involving Humans.

### Cell culture

#### PSC maintenance

PSCs and differentiations were cultured on 0.27 mg/mL diluted growth factor reduced Matrigel® (Corning, Cat#356231) or hESC-qualified Matrigel® (Fisher Scientific, Cat#08–774-552) diluted in DMEM/F12 (Thermo Fisher Scientific, Cat#11330032) according to the lot specific dilution factor. They were grown in mTeSR™1 medium (STEMCELL, Cat#85850) and expanded through routine aggregate passaging every 3–7 days using Gentle Cell Dissociation Reagent (STEMCELL, Cat#07174) to lift the cells, up to a maximum of 20 passages from the working cell bank.

#### Pancreatic progenitor differentiation

To initiate a differentiation experiment, PSC cultures were rinsed with 1 × Dulbecco′s Phosphate Buffered Saline without Mg^2+^ and Ca^2+^ (DPBS-) followed by incubation with Gentle Cell Dissociation Reagent for 8–10 min (min) at 37 °C. Released single cells were rinsed with DMEM/F12 with 15 mM HEPES (STEMCELL, Cat#36254) and spun at 300 rcf for 5 min. The resulting cell pellet was resuspended in mTeSR™1 medium supplemented with 10 μM Y-27632 (STEMCELL, Cat#72304) and the single cell suspension was seeded at 2.6 × 10^5^ cells/cm^2^ on 6- or 12-well plates coated for at least 1 h (h) at 37 °C with Matrigel®. Differentiation was initiated 24 h after seeding and according to the stepwise differentiation protocol of the STEMdiff™ Pancreatic Progenitor Kit (STEMCELL, Cat#05120). Unless otherwise stated, pancreatic progenitors were harvested at the end of the third day of Stage 4. Note that MEL1-INS^GFP/w^ cells in the static suspension culture experiments were differentiated in Stage 1 medium for three days instead of the recommended two by repeating the final day’s treatment (S1A for 1 day, S1B for two days). Following continued differentiation to Stage 4, they were harvested after four days in Stage 4 medium. During harvest, cells were treated for 12–15 min with ACCUTASE™ (STEMCELL, Cat#07920) at 37 °C. Released single cells were rinsed with DMEM/F12 and spun at 300 rcf for 5 min. The resulting cell pellet was resuspended in the S2-4 basal medium supplied with the STEMdiff™ Pancreatic Progenitor Kit. Following cell counting, cells were either aggregated or aliquoted for downstream analysis. Kit-derived pancreatic progenitors were compared to pancreatic progenitors generated using a previously published protocol^18^ with some modifications.

#### Cell aggregation

The single-cell suspension of pancreatic progenitors was aggregated using AggreWell™400 (STEMCELL, Cat#34415) or AggreWell™800 (STEMCELL, Cat#34815) plates. The plates were prepared prior to seeding following the manufacturer’s instructions. Unless otherwise stated, 750 cells/microwell were seeded when using AggreWell™400 plates and 3000 cells/microwell were seeded when using AggreWell™800 plates. Cells were aggregated in complete S4 medium + 10 μM Y-27632 for 24–48 h. If aggregating for 48 h, half the volume of spent medium was removed from each well after 24 h and gently replaced with a half volume of fresh medium to avoid displacement of aggregates. Notch inhibition during aggregation was done with 10 μM DAPT (STEMCELL, Cat#72082) for 48 h. Aggregates were harvested from plates by trituration and rinsed through a reversible 37 µm strainer (STEMCELL, Cat#27250) with DPBS- (Millipore Sigma, Cat#D8537). Aggregates were then washed out of the filter with DPBS- for analysis, or with Stage 5 medium for further differentiation in suspension culture. Aggregates were digested with ACCUTASE™ for 15 min at 37 °C to generate a single-cell suspension, which was analyzed for cell count and viability on a Nucleocounter® NC-3000™ Advanced Image Cytometer (ChemoMetec A/S) with AO/DAPI staining.

#### Transition of pancreatic progenitors into islet-like clusters in static suspension culture

The MEL1-INS^GFP/w^ cells used in these experiments were harvested at the end of Stage 4, day 4 and aggregated for 24 h at 3000 cells/aggregate. Kit-derived pancreatic progenitors were gently dislodged from AggreWell™ microwells and suspended in Stage 5 complete medium at a density of 10–20 clusters per 100 μL. Ultralow attachment flat bottom 96-well plates were first filled with 50 μL Stage 5 complete medium per well and then with 100 μL of the cluster resuspension as describe above in each well. The 96-well plate was placed on a level surface in an incubator at 5% CO2 at 37 °C. One hundred μL of spent medium was replaced with fresh media daily. For the induction of endocrine precursors, clusters were exposed to Stage 5 complete medium, which is made by supplementing S5-7 basal medium with 0.25 μM SANT-1 (Millipore Sigma, Cat#S4572), 0.05 μM retinoic acid (Millipore Sigma, Cat#R2625), 100 nM LDN193189 (STEMCELL, Cat#72147), 1 μM T3 (Millipore Sigma, Cat#T6397), 10 μM ALK5i II (Cayman Chemical Company, Cat#14794), 10 μM zinc sulfate (Sigma-Aldrich, Cat#Z0251), and 10 μg/mL heparin (Fisher Scientific, Cat#AC411210010) for 3 days. S5-7 basal medium consists of MCDB131 medium (Thermo Scientific, Cat#10372019) supplemented with 1.5 g/L NaHCO_3_ (Fisher Scientific, Cat#S233-500), 1% Glutamax (Gibco, Cat#35050061), 0.5% ITS-X (Thermo Fisher, Cat#51500056), 2% fatty acid-free bovine serum albumin (BSA; Proliant, Cat#7500804) and 15 mM glucose (Fisher Scientific, Cat#D14-212). For the induction of immature islets, clusters were exposed to Stage 6 complete medium (S5-7 basal medium supplemented with 100 nM LDN193189, 1 μM T3, 10 μM ALK5 inhibitor II, 100 nM gamma secretase inhibitor XX (Millipore Sigma, Cat#565789), 10 μM zinc sulfate and 10 μg/mL heparin) for 6–8 days. For the induction of maturing islets, clusters were exposed to Stage 7 complete medium (S5-7 basal medium supplemented with 1 μM T3, 10 μM ALK5 inhibitor II, 1 mM N-acetyl cysteine (Millipore Sigma, Cat#A9165), 10 μM Trolox (Cayman Chemical Company, Cat#10011659–250), 2 μM R428 (Cayman Chemical Company, Cat#21523–1), 10 μM zinc sulfate and 10 μg/mL heparin) for 8–12 days.

#### Stage 5 media comparisons

For the Stage 5 media testing, aggregates were added to 6-well plates (4 wells of AggreWell™400 plates combined per suspension well) and cultured on an INFORS HT Celltron orbital shaker at 80 rpm. Media volumes were 5 mL per well. Each day, 4 mL of spent media was replaced with fresh media to bring the volume up to 5 mL again. Rezania (2014) Stage 5 medium consisted of BLAR medium^18^ (HyClone, custom order) supplemented with 1.5 g/L NaHCO_3_, 1% Glutamax, 0.5% ITS-X, 2% fatty acid-free BSA, 20 mM glucose, 0.25 μM SANT-1, 0.05 μM retinoic acid, 100 nM LDN193189, 1 μM T3, 10 μM ALK5i II, 10 μM zinc sulfate, and 10 μg/mL heparin for 3 days. When indicated, 25 ng/mL FGF7 (STEMCELL, Cat#780046.1) was added for the duration of Stage 5 culture. Russ (2015) Stage 5 medium consisted of high glucose DMEM (STEMCELL, Cat#36250) supplemented with 1% B-27 (Gibco, Cat#17504044), 500 nM LDN193189, 1 μM ALK5i II, 25 ng/mL FGF7, and 30 nM TPB (EMD Millipore, Cat#565740). Nair (2019) Stage 5 medium consisted of high glucose DMEM supplemented with 1% Glutamax, 1% B-27, 0.5 mM vitamin C (Sigma Aldrich, Cat#A4544), 10 μg/mL heparin, 1% NEAA (Gibco, Cat#11140050), 500 nM LDN193189, 1 μM ALK5i II, 1 μM T3, and 1 μM gamma secretase inhibitor XX.

Whole well images were obtained using a custom STEMCELL imaging platform. Images were analyzed on CellProfiler (Broad Institute, Cambridge, MA) using a custom script (Supplemental File) after increasing the contrast and darkening the edges. The area of each detected aggregate is reported as the total number of pixels within and including the bounding box. The total aggregate area was calculated by the summation of all aggregate areas.

#### Human islet culture

Human islets were maintained in CMRL 1066 Supplemented, CIT modification medium (Corning, Cat# 98304CV) in an incubator at 5% CO_2_ at 37 °C. Media changes were performed every 1–2 days. Islets from healthy donors R356, R361 and R369 were used for static GSIS assays and measurement of total insulin content in this study. Details of islet donors are provided in Supplemental Table 2.

### Flow analysis

Single cell suspensions of cells were stained with LIVE/DEAD® Fixable Violet Dead Cell Stain (ThermoFisher Scientific, Cat# L34964) diluted in FACS Buffer (2% FBS in DPBS-) for 30 min. Cells were then fixed with Fixation/Permeabilization Solution (BD Biosciences, Cat#554714) for 10 min. Intracellular stains were then performed using various stains diluted in 1X Permeabilization/Wash Solution (BD Biosciences, Cat#554714) for 30–60 min. All incubation steps were performed at room temperature while protecting the samples from light. Dead cells were excluded during flow cytometry analysis and gating was determined using isotype antibodies. Refer to Supplemental Table 3 for antibody details. Flow analysis was performed using a Guava® easyCyte Instrument (Millipore Sigma) or Attune NxT Flow Cytometer (ThermoFisher Scientific). Flow cytometry data were analyzed and generated by FlowJo software v10.4 or higher (BD).

### Quantitative RT-PCR

Gene expression was assessed in differentiated cells following RNA isolation with a RNeasy® Mini Kit (Qiagen, Cat#74106), cDNA generation with an iScript™ gDNA Clear cDNA Synthesis Kit (Bio-Rad, Cat#1725035), and qPCR with the SsoFast™ EvaGreen® Supermix (Bio-Rad, Cat# 1725202). Data were normalized to human islet, pancreas, small intestine, or a control S5 sample, depending on the gene of interest and using NFX1 as a stably expressed reference gene. Refer to Supplemental Table 4 for primer details. Note that some data points are shared between Fig. 1E, 3E, and S5B to facilitate interpretation.

### Static GSIS assay

Static GSIS assays were performed as previously described with small modifications^49,50^. Briefly, clusters were rinsed once and equilibrated with low glucose KRB buffer (129 mM NaCl, 4.7 mM KCl, 2.5 mM CaCl_2_, 1.2 mM MgSO_4_, 1.2 mM KH_2_PO_4_, 5 mM NaHCO_3_, 10 mM HEPES, 0.1% BSA and 3.3 mM glucose) for 1 h at 37 °C in a 5% CO_2_ incubator. After equilibration, five clusters per group with replicates were incubated in low glucose KRB buffer for 30 min. The same clusters were then incubated in high glucose KRB buffer (129 mM NaCl, 4.7 mM KCl, 2.5 mM CaCl_2_, 1.2 mM MgSO_4_, 1.2 mM KH_2_PO_4_, 5 mM NaHCO_3_, 10 mM HEPES, 0.1% BSA and 16.7 mM glucose) for 30 min. The same clusters were finally incubated in depolarization KRB buffer (103.7 mM NaCl, 30 mM KCl, 2.5 mM CaCl_2_, 1.2 mM MgSO_4_, 1.2 mM KH_2_PO_4_, 5 mM NaHCO_3_, 10 mM HEPES, 0.1% BSA and 3.3 mM glucose) for 30 min. After each incubation supernatants were collected and stored at -30 °C. At the end of the experiment, clusters were immediately lysed for total insulin extraction. An insulin ELISA kit (ALPCO, Cat#80-INSHU-E01.1) was used to measure insulin content in the collected supernatants.

### Total insulin content

Islet clusters were suspended in acid–ethanol solution (1.5% HCl in 70% ethanol), manually pipetted, vigorously vortexed, and left at -30 °C overnight. The extracts were spun at 2000 g for 1 min, and supernatants were collected and neutralized with an equal volume of 1 M Tris–HCl (pH 7.5). An insulin ELISA kit (ALPCO, Cat#80-INSHU-E01.1) was used to measure insulin content in the collected supernatants. For data normalization in each experiment, an equal number of islet clusters from the same batch were washed with DPBS- and dissociated using Accutase for cell counting.

### Dithizone staining

Clusters were rinsed with DPBS- and then resuspended in 5 mg/mL dithizone (Sigma-Aldrich, Cat#194832) solution in DPBS- for 2–3 min at room temperature. The clusters were then washed thoroughly with DPBS- until the solution became colorless and transparent. Pictures were taken under a Axio Zoom.V16 microscope (Zeiss).

### Animal studies

Male NSG mice (NOD.Cg-Prkdcscid Il2rgtm1Wjl/SzJ, The Jackson Laboratory) were maintained on a 12-h light/dark cycle with ad libitum access to a standard irradiated diet (Teklad diet no.2918, Harlan Laboratories, Madison, WI). All experiments were carried out in accordance with the Canadian Council on Animal Care guidelines, were approved by the UBC Animal Care Committee, and reported following the recommendations in the ARRIVE guidelines^51^.

#### Implantation of hESC-derived pancreatic progenitor cells

Mice (8- to 10-week-old) were anesthetized with inhalable isoflurane and received implants of hESC-derived pancreatic progenitor cells under the left kidney capsule along with a single subcutaneous injection of enrofloxacin (Baytril, 10 mg/kg, Bayer Animal Health) as previously reported^52^. The cells were generated from two individual experiments, with the first going into cohort 1 and the second going into cohort 2 several weeks later. Mice received 3.2 million (cohort 1) or 4.9 million (cohort 2) cells per animal. In cohort 1, four mice received -DAPT cells and four mice received + DAPT cells. In cohort 2, five mice received -DAPT cells and seven mice received + DAPT cells. The sample size of each group was determined based on the available number of cells for implantation and on previous experience implanting pancreatic progenitors under the kidney capsule of mice. Mice were randomized into groups based on the standard “ = *RAND*()” function in Google Sheets. Progenitor cell aggregates were triturated out of the Aggrewell™ microwells, collected and washed through a reversible 37 µm filter with DPBS-, and spun at 100 rcf to pellet the cells in preparation for implantation. One well of the Aggrewell™ plate from each treatment group was harvested for cell counting prior to the procedure. The surgical procedure, monitoring, and metabolic tests were all performed in a Containment Level 1 animal facility and mice were acclimatized to handling for two weeks prior to surgery. All animals that underwent the surgical procedure were included in the study. Investigators were not blinded to the treatment groups. Euthanasia was performed at the experimental endpoint of 22–23 weeks post-implantation by isoflurane anesthesia and cervical dislocation. Kidneys were collected from euthanized mice and prepared for graft analysis.

#### Metabolic analysis

All metabolic analyses were performed in conscious, restrained mice on the indicated days. Testing order of cages was randomized each week. For all tests, blood glucose was measured via saphenous vein using a handheld glucometer (Lifescan; Burnaby, BC) and saphenous blood samples were collected at the indicated time points using heparinized microhematocrit tubes. Intraperitoneal glucose tolerance tests (IPGTTs) were performed after a 6-h fast and injection of glucose solution in sterile water (1 g/kg body weight). The arginine tolerance test (ArgTT) was performed following a 6-h fast and IP administration of arginine solution in sterile water (2 g/kg body weight). Blood glucose measurements and blood sample collections occurred at the indicated time points. Plasma was stored at − 30 °C and later assayed using a human C-peptide chemiluminescence ELISA (Alpco, Cat# 80-CPTHU-CH01) or a mouse C-peptide ELISA (Alpco, Cat# 80-CPTMS-E01). The primary outcome of the study was defined as detectable basal serum human C-peptide levels within 20 weeks of cell implantation. Secondary outcomes constituted an increase in human C-peptide levels in response to glucose or arginine challenges and maturation of implanted cells into endocrine, ductal, and exocrine tissues determined with immunofluorescent staining of the explanted grafts.

### Immunofluorescent staining

For paraffin sections, hESC-derived cells or mouse kidney explants were fixed for 1–2 days in 4% paraformaldehyde (PFA) at 4 °C. The hESC-derived cells were embedded in 1% agarose and kidneys were stored in 70% ethanol. Samples were then embedded in paraffin and sectioned (5 μm thickness; Wax-it Histology Services; Vancouver, Canada). Slides were deparaffinized and hydrated with xylenes and a graded alcohol series. Sections were subjected to 15 min of heat-induced epitope retrieval (HIER) at 95 °C using a 10 mM citrate buffer + 0.05% Tween 20, pH 6.0. Following a 10-min blocking step with Protein Block (Dako, Cat# X0909), slides were stained overnight at 4 °C with primary antibody diluted in Antibody Diluent (Dako, Cat# S3022) at the appropriate dilution. Following 3 washes with DPBS+ , sections were stained for 1 h at room temperature with secondary antibodies conjugated to AlexaFluor 488, 555, 594, or 647 as required. Following another 3 washes with DPBS+ , slides were counterstained and mounted using VECTASHIELD® HardSet™ Antifade Mounting Medium with DAPI (Vector Laboratories, Cat# H-1500). Antibodies used are detailed in Supplemental Table 5. Whole slide immunofluorescence scanning was performed using an ImageXpressMicro™ Imaging System (Molecular Devices Corporation, Sunnyvale, CA), and images were stitched together and analyzed using MetaXpress Software. The total number of positive and negative stained cells were counted in the hESC-derived graft. The ratio of individual positive stained cells to total positive stained cells was then calculated for each graft.

For whole-mount immunostaining, clusters were fixed overnight in 4% PFA at 4 °C, rinsed with 0.1% Triton-X in DPBS + (PBST), and permeabilized in 0.3% Triton-X in DPBS + for 6–16 h. Samples were then blocked for 1–2 h with 5% BSA in 0.3% Triton-X and stained with primary antibodies diluted in 1% BSA in 0.3% Triton-X overnight. Aggregates were washed three times with PBST at 30-min intervals before secondary staining overnight in 1% BSA in 0.3% Triton-X. After a brief wash with PBST, nuclei were stained with 10 μg/mL DAPI (Sigma, Cat# D9542) in PBST for 10–15 min and then washed three times with PBST at 30-min intervals. A tilt shaker was utilized for gentle agitation at room temperature during the incubation steps following fixation. Clusters were first attached on confocal chamber slides using Cell-Tak tissue adhesive (Corning, Cat# 354240) and then mounted with tissue clearing solution (80% glycerol in DPBS +) and covered with coverslips. Immunofluorescence imaging was performed using a Leica TCS SP5 X laser scanning confocal microscope (Leica Microsystems, Wetzlar, Germany) and images were analyzed using Fiji software.

### Statistical analysis

All statistics were performed using GraphPad Prism 9 software (GraphPad Software Inc., La Jolla, CA). Specific statistical tests for each experiment are described in the figure legends. Area under the curve (AUC) was calculated using the fasting blood glucose level for each mouse as the baseline. For all analyses, *P* < 0.05 was considered statistically significant. Bar chart data are presented as box and whisker, floating bar, mean ± SD, or mean ± SEM with individual biological replicates shown as separate data points.

## Supplementary Information

Supplementary Information 1.

Supplementary Information 2.

## Data Availability

The datasets generated and/or analysed in this manuscript are not publicly available due to a collaboration with STEMCELL Technologies but are available from the corresponding author on reasonable request.
