# Supplementary figures and images for: Protocol development to further differentiate and transition stem cell-derived pancreatic progenitors from a monolayer into endocrine cells in suspension culture
Source: Sci Rep. 2023 Jun 1;13:8877. doi: 10.1038/s41598-023-35716-1 (PMC10235054; doi:10.1038/s41598-023-35716-1)

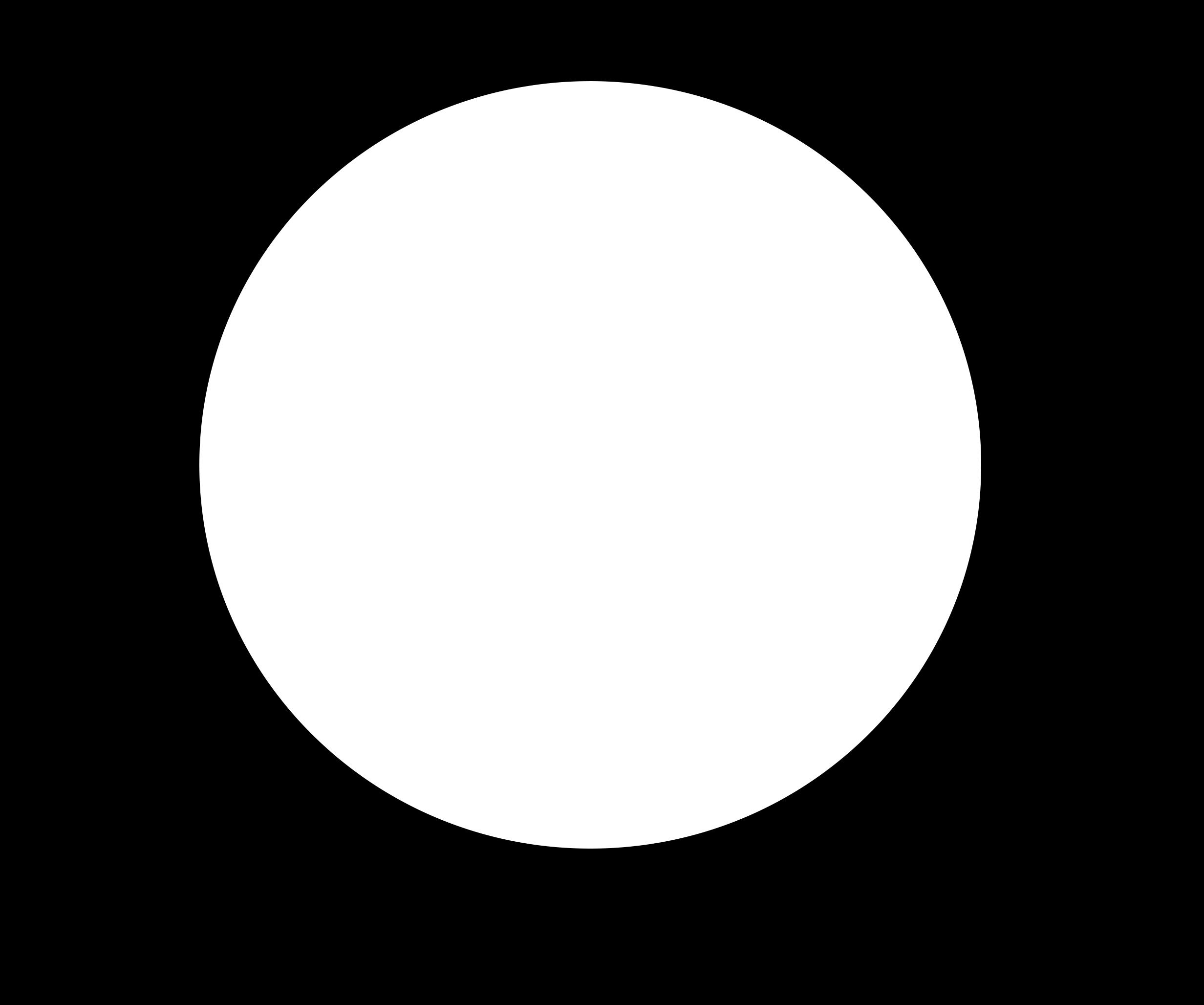

Supplement: Supplementary file 1 — Supplementary Information 1. [file 41598_2023_35716_MOESM1_ESM.zip › PlateTemplate.png]
