# Supplementary material for: Protocol development to further differentiate and transition stem cell-derived pancreatic progenitors from a monolayer into endocrine cells in suspension culture
Source: Sci Rep. 2023 Jun 1;13:8877. doi: 10.1038/s41598-023-35716-1 (PMC10235054; doi:10.1038/s41598-023-35716-1)
Supplement: Supplementary file 2 — Supplementary Information 2. [file 41598_2023_35716_MOESM2_ESM.pdf]

SUPPLEMENTAL FIGURES

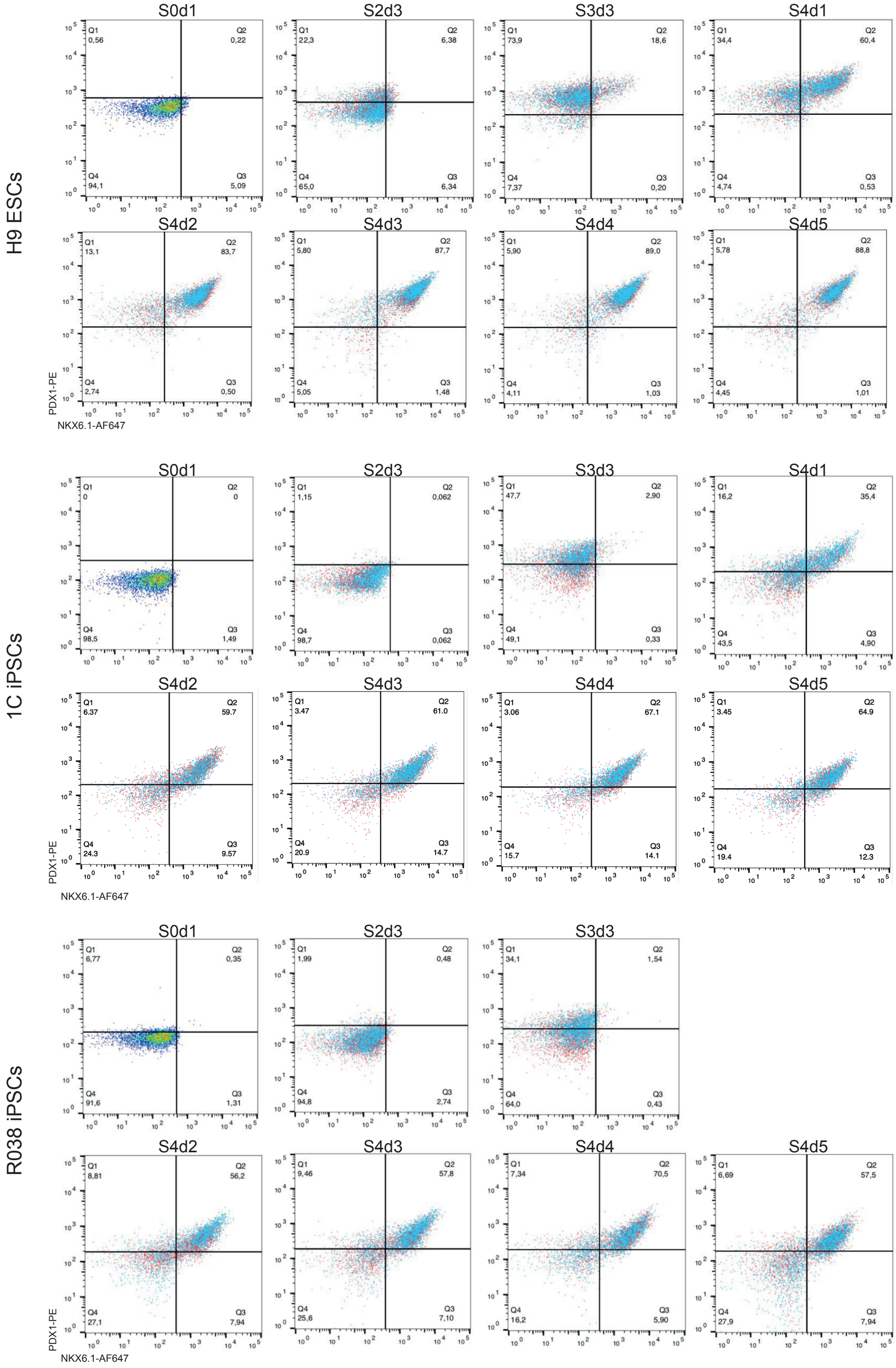

Figure S1. Flow cytometry analysis for PDX1 and NKX6.1 at various time points of kit pancreatic progenitor differentiation in H1, 1C, and R038 PSCs, related to Figure 1.
Except for S0d1 data, all timepoints are shown with two biological replicates differentiated in separate wells during the same experiment (blue and red dots on flow plot).

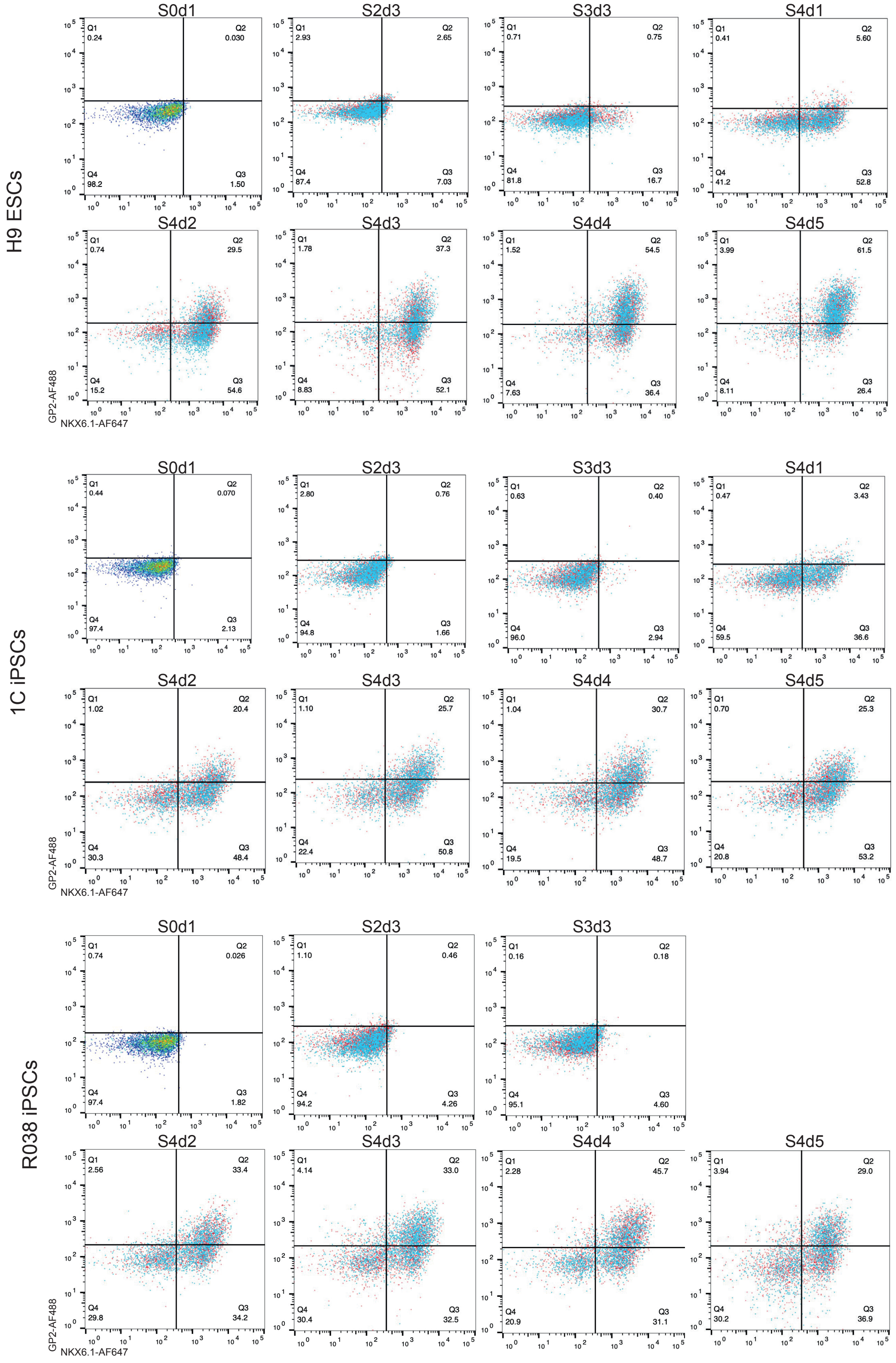

Figure S2. Flow cytometry analysis for GP2 and NKX6.1 at various time points of kit pancreatic progenitor differentiation in H1, 1C, and R038 PSCs, related to Figure 1.
Except for S0d1 data, all timepoints are shown with two biological replicates differentiated in separate wells during the same experiment (blue and red dots on flow plot).

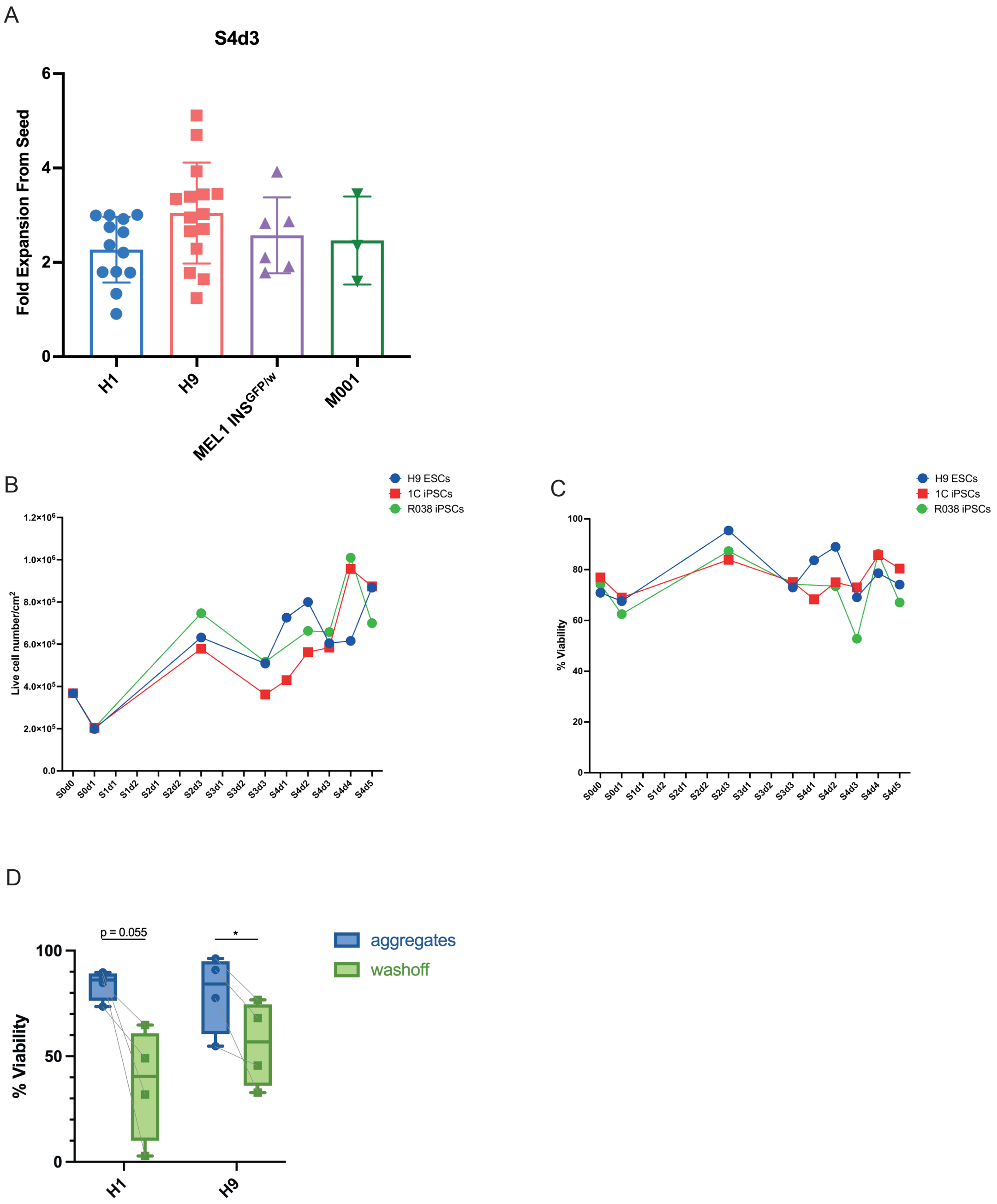

Figure S3. Further characterization of kit-derived pancreatic progenitors pre- and post-aggregation, related to Figure 1.

(A) Fold expansion reported at Stage 4, day 3 relative to the initial seed number. Data are presented as mean ± SD.

(B) Cell counts from three PSC lines at various time points of kit pancreatic progenitor differentiation.

(C) Cell viability from three PSC lines at various time points of kit pancreatic progenitor differentiation.

(D) Cell viability from two PSC lines comparing cells that are retained in the aggregates rinsing through a 37 μm reversible strainer and cells that washed off the aggregate and through the strainer (n = 4 independent experiments). *$p<0.05$ by paired t-test.

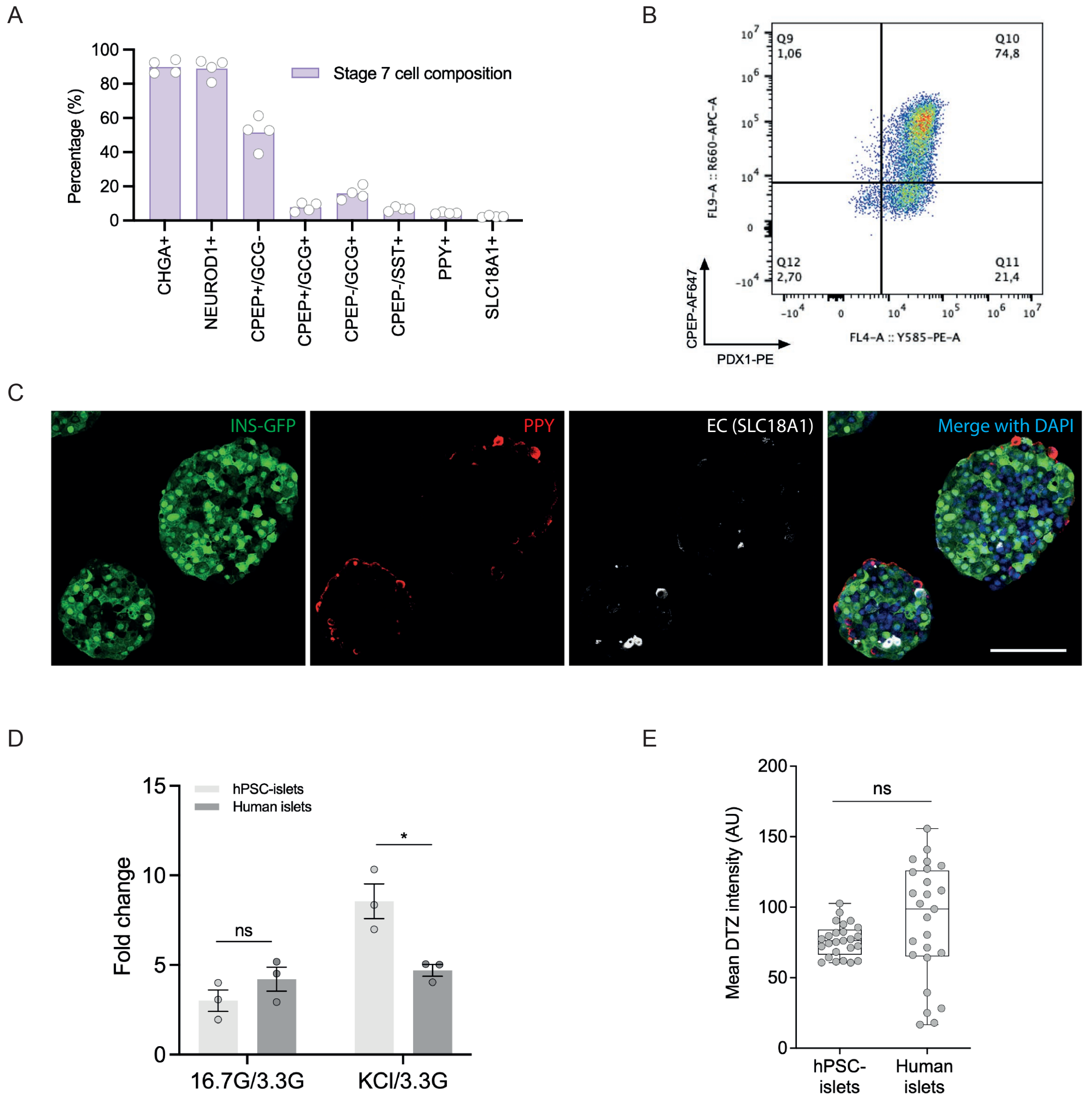

Figure S4. Further characterization of MEL1-INS$^{GFP/w}$ Stage 7 clusters, related to Figure 2.

(A) Summary of whole mount immunostaining data from 4 independent differentiations.

(B) Representative flow cytometry analysis for C-peptide (CPEP) and PDX1 in Stage 7 hPSC-islets.

(C) Representative whole-mount immunostaining of the INS^{GFP} signal (green); pancreatic polypeptide, PPY (red); enterochromaffin cell (EC) SLC18A1 (gray); DAPI (blue). Scale bar, 100 μm.

(D) Static GSIS assays showing fold change in insulin secretion from Stage 7 hPSC-islets and primary human islets (n = 3 independent experiments) in response to low glucose (3.3G, 3.3 mM glucose), high glucose (16.7G, 16.7 mM glucose), and 30 mM KCl depolarization challenge. Data are presented as mean ± SEM. *$p<0.05$ by unpaired two-tailed t-test.

(E) Quantification of mean dithizone (DTZ) intensity in the DTZ-stained Stage 7 hPSC-islets and the typical human islet preparation in Figure 2I. No significance calculated by unpaired two-tailed t-test.

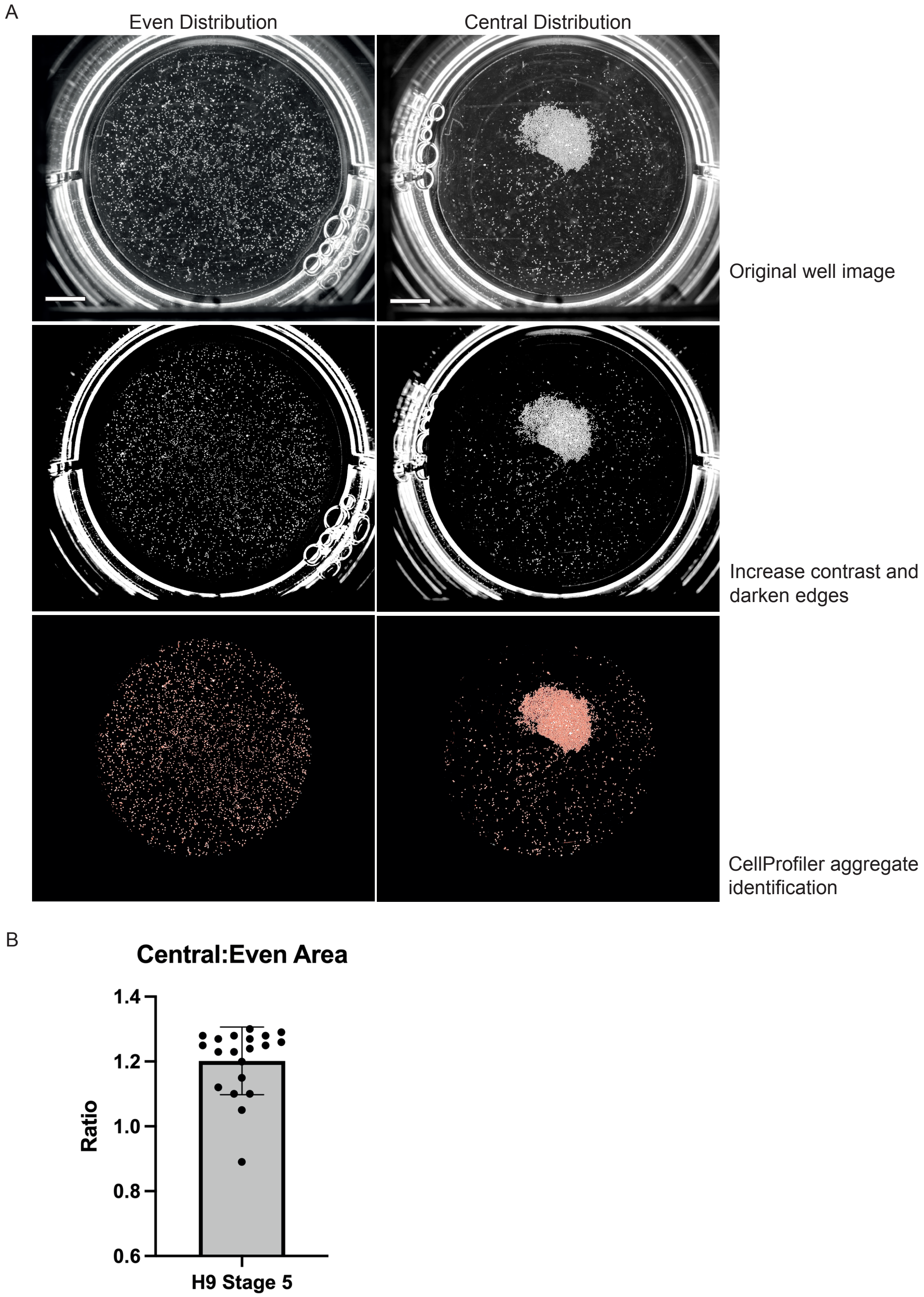

Figure S5. Whole-well imaging of suspension culture wells can be used to monitor aggregate survival over time, related to Figure 3.

(A) Representative images of aggregates in 6-well suspension culture. After increasing the contrast and darkening the edges, the CellProfiler script was able to identify most aggregates within the field of view from evenly and centrally distributed spheroids. Scale bar = 50 mm.

(B) The ratio of the total area of aggregates reported for evenly and centrally distributed aggregates from the same wells (n = 20 wells imaged). Data are presented as mean ± SD.

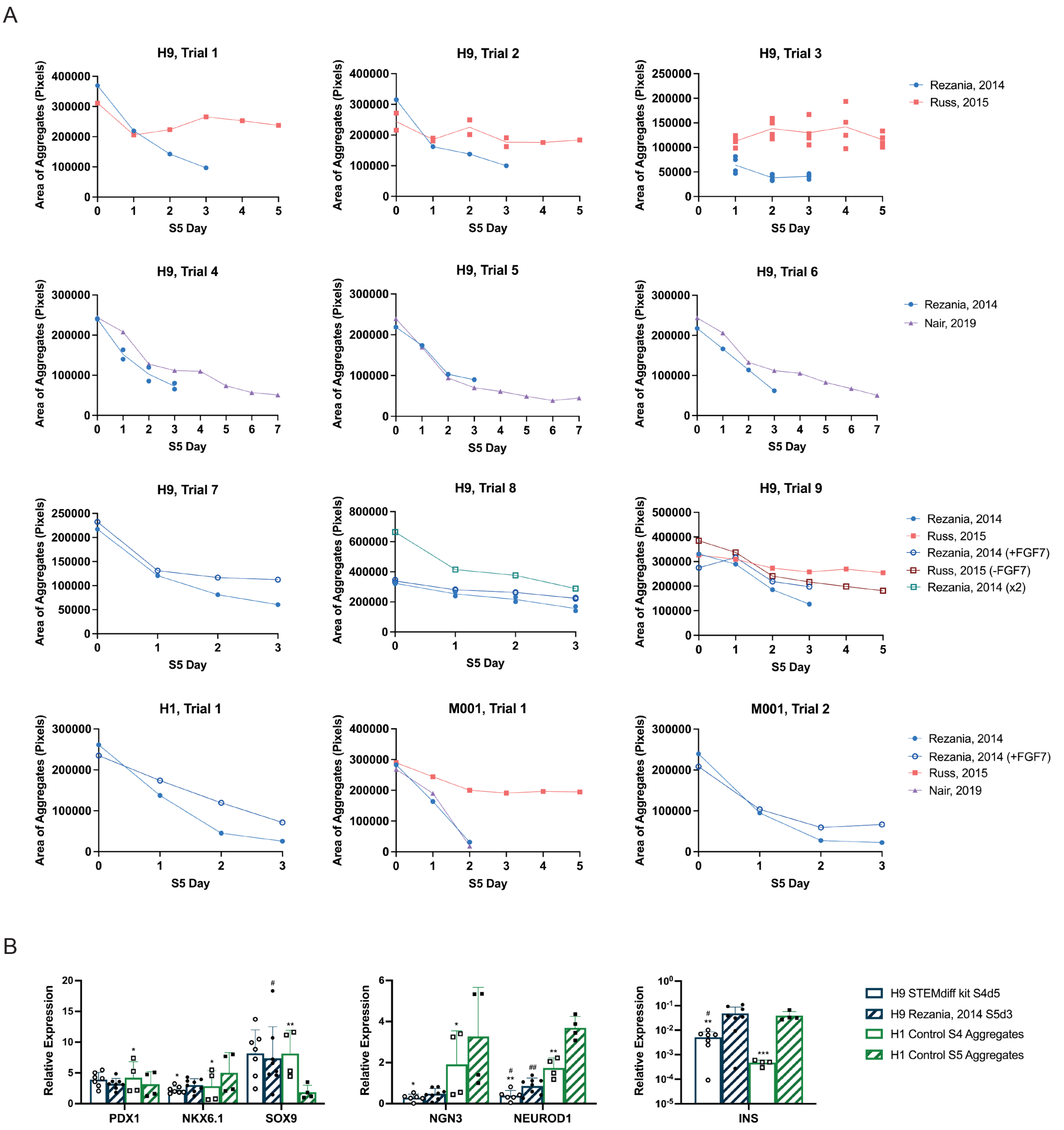

Figure S6. Further characterization of aggregated pancreatic progenitors as they progress through stage 5 suspension culture, related to Figure 3.

(A) Individual trial quantification of aggregate area every day during S5 culture relative to the initial seed (n = 1-4 wells per condition). Each trial represents an independent differentiation experiment. In cases where multiple wells were used for a condition, data from each well is shown. In Trial 7 using the H9 cells, we tested adding twice the usual number of aggregates to one of the wells.

(B) Gene expression analysis of kit-derived H9 stage 4 aggregates, subsequent S5 aggregates using the Rezania formulation, and H1 stage 4 and stage 5 aggregates using our control protocol (n = 4-8 independent experiments). *PDX1*, *NKX6.1*, *NEUROD1*, and *INS* are displayed relative to human islet, *SOX9* is displayed relative to whole human pancreas, and *NGN3* is displayed relative to a control stage 5 differentiation. Note that some data points are shared with Figures 1E and 3E to facilitate interpretation. Data are presented as mean ± SD. * $p<0.05$; ** $p<0.005$; *** $p<0.0005$ by ratio paired t-test comparing stage 4 to stage 5 from the same experiment. # $p<0.05$; ## $p<0.005$ by unpaired two-tailed t-test with Welch correction comparing stage 4 or stage 5 between the two conditions.

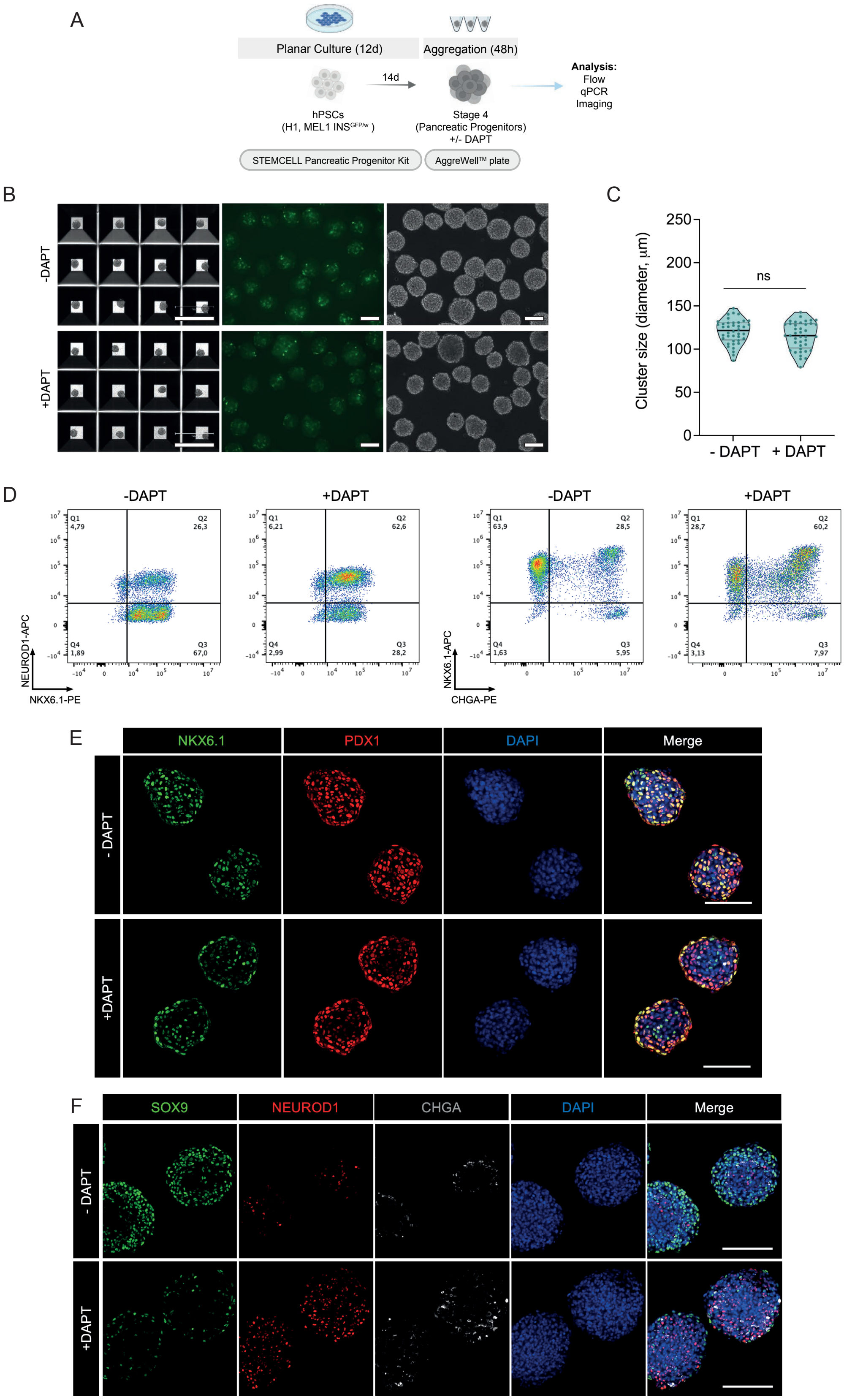
A
Planar Culture (12d)
Aggregation (48h)
14d
hPSCs
(H1, MEL1 INSGFP/w)
Stage 4
(Pancreatic Progenitors)
+/- DAPT
Analysis:
Flow
qPCR
Imaging
STEMCELL Pancreatic Progenitor Kit
AggreWellTM plate
B
-DAPT
+DAPT
C
Cluster size (diameter, μm)
ns
- DAPT
+ DAPT
D
-DAPT
+DAPT
NEUROD1-APC
NKX6.1-PE
NKX6.1-APC
CHGA-PE
Q1 4,79
Q2 26,3
Q3 67,0
Q4 1,89
Q1 6,21
Q2 62,6
Q3 28,2
Q4 2,99
Q1 63,9
Q2 28,5
Q3 5,95
Q4 1,63
Q1 28,7
Q2 60,2
Q3 7,97
Q4 3,13
E
NKX6.1
PDX1
DAPI
Merge
- DAPT
+DAPT
F
SOX9
NEUROD1
CHGA
DAPI
Merge
- DAPT
+DAPT

Figure S7. Further characterization of Notch-inhibition of MEL1-INS$^{GFP/w}$ pancreatic progenitors during aggregation, related to Figure 4.
(A) Differentiated pancreatic progenitors were harvested and aggregated with or without DAPT treatment for 48 h prior to analysis. Cells aggregated at 750 cells/cluster (B-D) or 3000 cells/cluster (E) for 48 hours in AggreWell™800 plates.
(B) Phase contrast (left, right) and GFP fluorescence (middle) images. Scale bars of left column = 750 µm, scale bars of center column and right column = 100 µm.
(C) Quantification of cluster size at indicated stages (n = 2 independent experiments). No significance calculated by unpaired two-tailed t-test.
(D) Representative flow cytometry analysis for NKX6.1, NEUROD1, and CHGA.
(E) Representative whole-mount immunostaining images. NKX6.1 (green); PDX1 (red); DAPI (blue). Scale bar = 100 µm.
(F) Representative whole-mount immunostaining images. SOX9 (green); NEUROD1 (red); Chromogranin A, CHGA (grey); DAPI (blue). Scale bar = 100 µm.

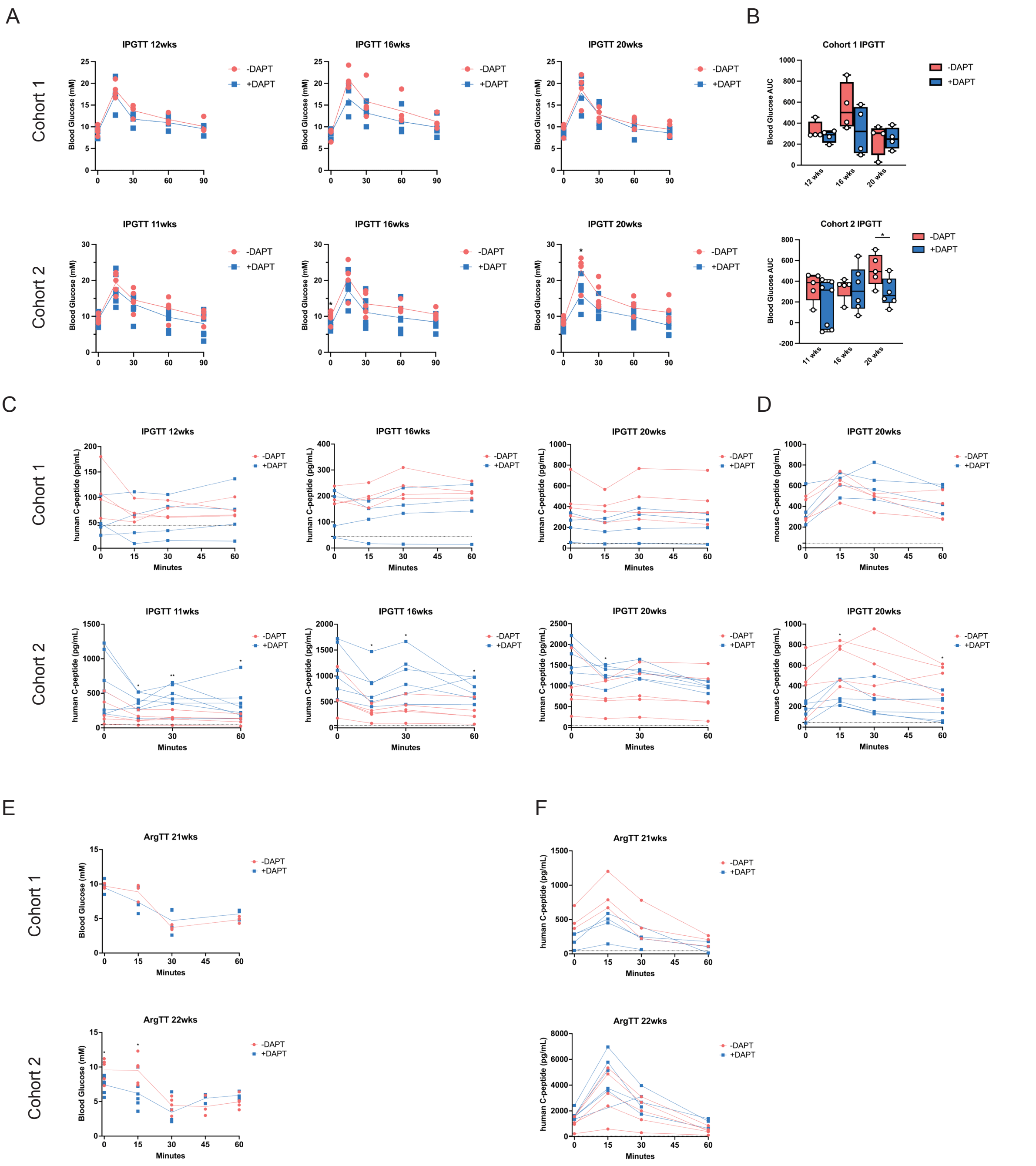

Figure S8. Further analysis of IPGTT and ArgTT testing in both mouse cohorts, related to Figure 5.

(A) Blood glucose monitoring from IPGTTs 11-20 weeks post-implant.
(B) AUC analysis of blood glucose clearance from above IPGTTs.
(C) Human C-peptide levels measured from serum collected during above IPGTTs.
(D) Mouse C-peptide levels measured from serum collected during IPGTTs 20-weeks post-implant.
(E) Blood glucose monitoring from ArgTTs 21-22 weeks post-implant.
(F) Human C-peptide levels measured from serum collected during above ArgTTs.
n = 4-7 mice per treatment group per cohort. * $p<0.05$ by unpaired two-tailed t-test with Welch correction comparing the two treatment groups at each timepoint.

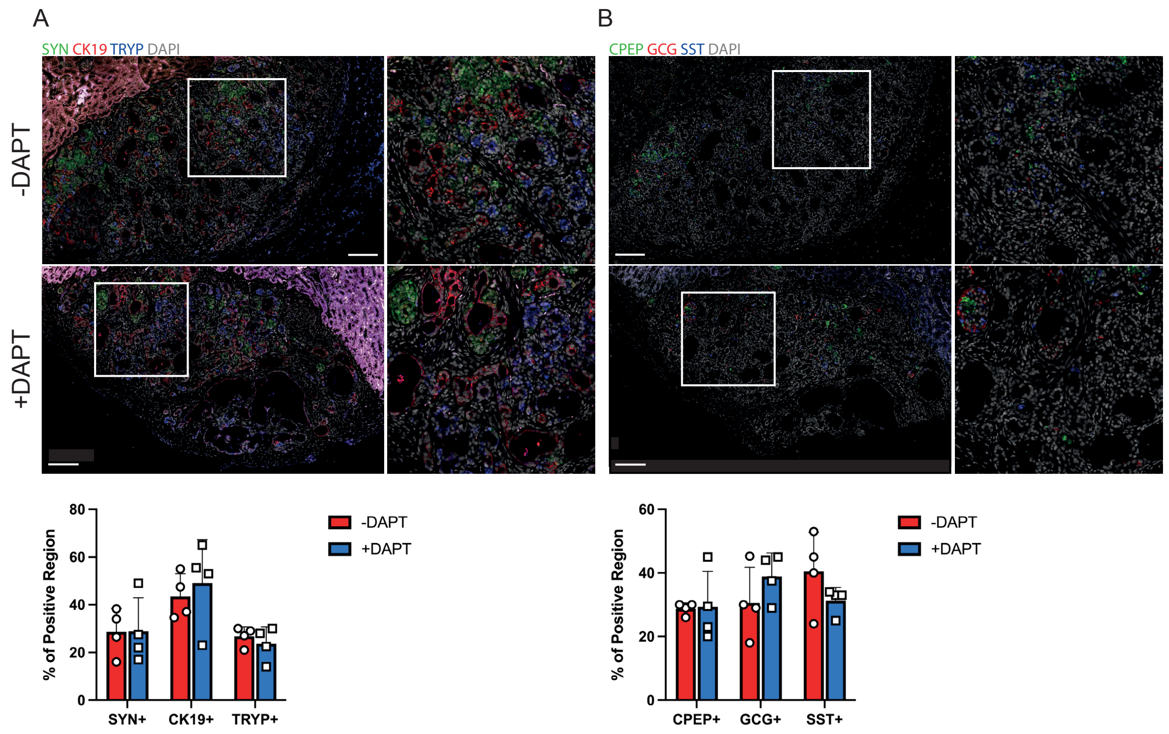

Figure S9. Analysis of explanted grafts from cohort 1, related to Figure 5.
(A, B) Immunohistochemical staining of the explanted grafts of cohort 1 (n = 4 mice per treatment group). Stains were for synaptophysin (SYN), CK19, and trypsin (TRYP) or C-peptide (CPEP), glucagon (GCG), and somatostatin (SST). A representative graft from each treatment group is shown as well as the summary data from all mice. Scale bar = 500 μm. No significance was detected between treatment groups by unpaired two-tailed t-test with Welsh correction.

Tables

Supplemental Table 1. Medium formulations tested in Stage 5 culture

**Rezania, 2014**	**Russ, 2015**	**Nair, 2019**
BLAR medium	DMEM	DMEM
+1.5 g/L sodium bicarbonate		
1X Glutamax		1X Glutamax
20 mM glucose	25 mM glucose	25 mM glucose
2% BSA	1:100 B27	1:100 B27
1:200 ITS-X		
10 μM zinc sulfate		10 μM zinc sulfate
10 μg/ml heparin		10 μg/ml heparin
		500 μM Vitamin C
		1:100 NEAA
		1 mM N-acetyl cysteine
	25 ng/mL FGF7	
0.25 μM SANT-1		
0.05 μM RA		
100 nM LDN193189	500 nM LDN193189	500 nM LDN193189
	30 nM TPB	
1 μM T3		1 μM T3
10 μM ALK5iII	1 μM ALK5iII	10 μM ALK5iII
		1 μM XXi

RA, retinoic acid; T3, Triiodothyronine; TBP, PKC activator V; XXi, NEAA, non-essential amino acids; γ-secretase inhibitor XXi

Supplemental Table 2. Human islet donors used in this study

Donor ID	Age (years)	Sex	BMI	HbA1c (%)	Cold Ischemic Time (h)
R356	45	Female	29.7	5.1	14.0
R361	65	Female	20.8	5.3	17.5
R369	66	Male	25.6	4.9	12.0

Supplemental Table 3. Antibody information for flow analysis

Conjugated Target Antibody	Cat #	Dilution	Conjugated Isotype Antibody	Cat #	Dilution
PE Mouse Anti-PDX-1	BD 562161	1:33	PE Mouse IgG1, κ Isotype Control	BD 554680	1:561
Alexa Fluor® 647 Mouse Anti-Nkx6.1	BD 563338	1:33	Alexa Fluor® 647 Mouse IgG1 κ Isotype Control	BD 557732	1:17
PE Mouse Anti-Glucagon	BD 565860	1:2000	PE Mouse IgG1, κ Isotype Control	BD 554680	1:2000
Alexa Fluor® 647 Mouse Anti-C-Peptide	BD 565831	1:2000	Alexa Fluor® 647 Mouse IgG1 κ Isotype Control	BD 557714	1:50
PE Mouse Anti-NeuroD1	BD 563001	1:33	PE Mouse IgG1, κ Isotype Control	BD 554680	1:561
Alexa Fluor® 647 Mouse Anti-Sox9	BD 565493	1:33	Alexa Fluor® 647 Mouse IgG1 κ Isotype Control	BD 557714	1:17
Anti-GP2 mAb-Alexa Fluor® 488	MBL D277-A48	1:1000	Mouse IgG1 Alexa Fluor® 488	MBL M075-A48	1:1000
Alexa Fluor® 647 Mouse Anti-NeuroD1	BD 563566	1:50	Alexa Fluor® 647 Mouse IgG1 κ Isotype Control	BD 557714	1:50
PE Mouse Anti-Nkx6.1	BD 563023	1:250	PE Mouse IgG1, κ Isotype Control	BD 554680	1:2000
PE Mouse Anti-Human Chromogranin A	BD 564563	1:200	PE Mouse IgG1, κ Isotype Control	BD 554680	1:2000
Alexa Fluor® 488 Mouse Anti-PDX-1	562274	1:33	Alexa Fluor® 488 Mouse IgG1, κ Isotype Control	BD 565572	1:300

Green and blue colored isotype antibodies represent different catalog numbers of the same conjugated isotype, which have different stock concentrations.

Supplemental Table 4. List of primers used for quantitative RT-PCR analysis

**Targeted cDNA**	**Fwd primer (5' --> 3')**	**Rev primer (5' --> 3')**	**Transcript Accession #**	**Amplicon size**
PDX1	CCCTCTTTTAGTGATACTGGATTGG	CCTTCCAATGTGTATGGTACAGTTTC	ENST00000381033.4	141 bp
NKX6.1	CCTGTACCCCTCATCAAGGAT	CAAGTATTTTGTTTGTTCGAAAGTCTTCT	ENST00000295108.3	125 bp
NGN3	ACCACCCCATAATCTCATTCAAAG	GTAAGAGACTGAGAGGCAGACAG	ENST00000242462.5	117 bp
SOX9	CTGGGCAAGCTCTGGAG	CGTTCTTCACCGACTTCCTC	NM_000346.4	139 bp
CDX2	GAGTTTCACTACAGTCGCTACATCA	GCTGCAACTTCTTCTTGTTGATTTTC	ENST00000381020.7	142 bp
NEUROD1	GGTTATGAGACTATCACTGCTCAG	AGAACTGAGACACTCGTCTGTC	ENST00000295108.3	140 bp
INS	GCAGCCTTTGTGAACCAACA	GGTGTGTAGAAGAAGCCTCGTT	NM_000207.2	89 bp
HES1	CATCTGAGCACAGAAAGTCATCAAAG	GCTTCACTGTCATTTCCAGAATGTC	ENST00000232424.4	168 bp
NFX1	TTTCAGAACAAAGGAGCTTCCAT	CTTATCCACACAGCATATCTCATTACA	ENST00000379540.7	130 bp

Supplemental Table 5. Antibody information for immunofluorescent staining

**Primary Stains**				
**Antigen**	**Species**	**Cat #**	**Dilution (whole-mount dilution)**	
PDX1	Rabbit	Abcam ab47267	(1:200)	
NKX6.1	Mouse	DSHB F55A12-c	(1:50)	
Glucagon (GCG)	Mouse	Sigma G2654	1:1000 (1:400)	
Somatostatin (SST)	Rabbit	Sigma HPA019472	1:500 (1:100)	
SOX9	Rabbit	Millipore AB5535	(1:200)	
NEUROD1	Goat	R&D AF2746	(1:20)	
Synaptophysin (SYN)	Rabbit	Novus NB120-16659	1:50	
Cytokeratin 19 (CK19)	Mouse	Dako M0888	1:100	
Trypsin (TRYP)	Sheep	R&D AF3586	1:25	
C-peptide (CPEP)	Guinea Pig	Abcam ab30477	1:100	
Chromogranin A (CHGA)	Mouse	Dako M0869	(1:100)	
SLC18A1	Rabbit	Sigma HPA063797	(1:300)	
Pancreatic Polypeptide (PPY)	Goat	R&D AF6297	(1:100)	
**Secondary Stains**				
**Antigen**	**Species**	**Conjugated Fluorophore**	**Cat #**	**Dilution (whole-mount dilution)**
Goat	Donkey	AF555	Life Technologies A21432	(1:500)
Mouse	Donkey	AF488	Life Technologies A31572	(1:500)
Mouse	Donkey	AF555	Life Technologies A31570	1:1000 (1:500)
Mouse	Donkey	AF647	Life Technologies A31571	(1:500)
Rabbit	Donkey	AF488	Life Technologies A21206	1:1000
Rabbit	Donkey	AF555	Life Technologies A11073	(1:500)
Rabbit	Donkey	AF647	Life Technologies A31573	(1:500)
Sheep	Donkey	AF647	Life Technologies A21448	1:1000
Guinea Pig	Goat	AF488	Life Technologies A11073	1:1000
Mouse	Goat	AF555	Life Technologies A21424	1:1000
Rabbit	Goat	AF647	Life Technologies A21245	1:1000
Rabbit	Goat	AF555	Life Technologies A21429	1:1000

Antibody dilutions used for whole-mount immunostaining differ from the dilutions used for section immunostaining and are indicated with parentheses.
